# Association of Metallic and Nonmetallic Elements with Fibrin Clot Properties and Ischemic Stroke

**DOI:** 10.3390/life14050634

**Published:** 2024-05-16

**Authors:** Hieronim Jakubowski, Marta Sikora, Ewa Bretes, Joanna Perła-Kaján, Olga Utyro, Izabela Wojtasz, Radosław Kaźmierski, Marcin Frankowski, Anetta Zioła-Frankowska

**Affiliations:** 1 Department of Microbiology, Biochemistry & Molecular Genetics, Rutgers-New Jersey Medical School, International Center for Public Health, 225 Warren Street, Newark, NJ 07103, USA; 2 Department of Biochemistry and Biotechnology, Poznań University of Life Sciences, 60-632 Poznań, Poland; ewa.bretes@up.poznan.pl (E.B.); joanna.kajan@up.poznan.pl (J.P.-K.); olga.utyro@gmail.com (O.U.); 3 European Center for Bioinformatics and Genomics, Institute of Bioorganic Chemistry, 61-704 Poznań, Poland; mperdziak@yahoo.com; 4 Medicover, 61-894 Poznań, Poland; iza.wojtasz@gmail.com; 5 Department of Neurology, Collegium Medicum, University of Zielona Góra, 65-046 Zielona Góra, Poland; rkazmierski@ump.edu.pl; 6 Department of Neurology, Poznań University of Medical Sciences, 61-701 Poznań, Poland; 7 Faculty of Chemistry, Adam Mickiewicz University, 61-614 Poznań, Poland; marcin.frankowski@amu.edu.pl (M.F.); anetta.ziola-frankowska@amu.edu.pl (A.Z.-F.)

**Keywords:** metals, nonmetals, fibrin clot properties, ischemic stroke

## Abstract

Objectives—Metallic elements and fibrin clot properties have been linked to stroke. We examined metallic and nonmetallic elements, fibrin clot lysis time (CLT), and maximum absorbance (Abs_max_) in relation to ischemic stroke. Design—A case–control study of ischemic stroke patients vs. healthy individuals. Subjects and Methods—Plasma and serum were collected from 260 ischemic stroke patients (45.0% women; age, 68 ± 12 years) and 291 healthy controls (59.7% women; age, 50 ± 17 years). Fibrin CLT and Abs_max_ were measured using a validated turbidimetric assay. Serum elements were quantified by inductively coupled plasma mass spectrometry (ICP-MS) and optical emission spectrometry (ICP-OES). Data were analyzed by bivariate correlations and multiple or logistic regression. Results—In female stroke patients, copper, lithium, and aluminum were significantly lower compared with controls; in male stroke patients, potassium was lower, and beryllium was elevated. In female and male stroke patients, iron, zinc, nickel, calcium, magnesium, sodium, and silicon were significantly lower, while strontium was elevated. Positive correlations between fibrin clot properties and metals, observed in healthy controls, were lost in ischemic stroke patients. In multivariate regression analysis, fibrin CLT and/or Abs_max_ was associated with zinc, calcium, potassium, beryllium, and silicon in stroke patients and with sodium, potassium, beryllium, and aluminum in controls. In logistic regression analysis, stroke was independently associated with lithium, nickel, beryllium, strontium, boron, and silicon and with sodium, potassium, calcium, and aluminum but not with fibrin CLT/Abs_max_. Conclusions—Various elements were associated with fibrin clot properties and the risk of ischemic stroke. Lithium, sodium, calcium, and aluminum abrogated the association of fibrin clot properties with ischemic stroke.

## 1. Introduction

Stroke is the second leading cause of morbidity and mortality in the world [1] with the overall burden increasing across the globe in both men and women of all ages [2]. Ischemic stroke causes an injury in the brain that is mediated by thrombotic or embolic events originating from a cardiac source or periphery [3]. Thrombus formation involves the generation of a fibrin mesh scaffold that involves complex interactions between components of the coagulation cascade [4]. Although traditional (hypertension, diabetes, obesity, dyslipidemia, and glycemia [5]) and nontraditional risk factors, e.g., hyperhomocysteimia [6], for stroke are known, these factors do not accurately predict vascular events.

While accumulating evidence suggests that fibrin clot properties are associated with the development and progression of cardiovascular disease (CVD) [4,7,8], the available data for fibrin clot properties in acute ischemic stroke are limited. For example, altered fibrin clot structure and function, reflected in increased maximum absorbance (Abs_max_) and longer clot lysis time (CLT), respectively, have been reported in stroke patients [9,10]. However, another study reported that prolonged CLT did not affect the risk of stroke in young women, whereas shorter CLT increased the risk [11]. Nevertheless, understanding factors that affect fibrin clot properties and the risk of ischemic stroke is important in the development of preventive and treatment strategies [12].

Trace elements such as iron (Fe), cobalt (Co), copper (Cu), zinc (Zn), manganese (Mn), molybdenum (Mo), vanadium (V), selenium (Se), tin (Sn), iodine (I), fluorine (F), and silicon (Si) are essential for human physiology [13]. However, essential trace elements can be toxic when the limit of safe exposure is exceeded. Metals of hydrogeological origin present in the environment (arsenic (As), lead (Pb), cadmium (Cd), mercury (Hg), and Cu, reviewed in ref. [14]; As, Pb, Cd, Hg, Cu, barium (Ba), chromium (Cr), nickel (Ni), Se, and Zn, reviewed in ref. [15]) are inherently toxic and have been linked to increased risk of CVD and stroke. Specifically, elevated serum Cu is associated with CVD mortality [16], acute myocardial infarction (AMI), and stroke [17]. Environmental exposure to Ni (assessed by analysis of urine) has been linked to a higher incidence of CVD in US adults independent of traditional risk factors [18]. In a Chinese population, exposure to Cd, As, Ni, aluminum (Al), and Cu was positively associated with diabetes, and exposure to Cd was associated with stroke [19]. In another study from China, urinary metals (Co, Cr, Ni, Cd, As, Fe, Zn, and Pb) were associated with the risk of hypertension [20].

Alterations in the homeostasis of essential elements have been linked to CVD and stroke. For instance, low plasma sodium (Na) levels are a risk factor for stroke and CVD [21]. Plasma Cu, Mo, and titanium (Ti) were associated with a higher risk of ischemic stroke, while rubidium (Rb) and Se were associated with a lower risk of hemorrhagic stroke in a Chinese population [22]. Another study of a Chinese population found that higher plasma concentrations of Al, As, and Cd and lower concentrations of Fe and Se were associated with an increased risk of ischemic stroke [23]. Alterations in calcium (Ca) homeostasis are associated with human diseases, including stroke [24]. The ingestion of potassium (K)-rich foods reduces, while insufficient K intake increases, the incidence of stroke and CVD [25]. Higher and lower Na excretion are significantly associated with an increased risk of stroke [26]. Lower plasma magnesium levels may contribute to a higher risk of ischemic stroke among women [27]. Serum Zn levels are negatively associated with ischemic stroke incidence, especially among women [28]. Nonmetallic elements such as phosphorus [29] and boron [30] have also been linked to ischemic stroke.

How essential and nonessential elements affect fibrin clot structure and function in relation to stroke is not known. The present study was designed to examine serum metallic and nonmetallic elements (Li, Ni, Be, Sr, B, S, and Si) that are not known to be associated with ischemic stroke and fibrin clot properties. We also examined serum Fe, Cu, Zn, Ca, Sr, Mg, Na, K, Al, and P, which were previously reported to be associated with ischemic stroke but are not known to be associated with fibrin clot properties. The associations of these elements with fibrin clot properties and the incidence of ischemic stroke were studied in a Polish population. We also studied determinants of CLT and Abs_max_ in stroke patients and healthy individuals. Possible interactions between individual elements and fibrin clot properties as determinants of ischemic stroke were also examined.

## 2. Materials and Methods

### 2.1. Participants

Participants included consecutive ischemic stroke patients from the Poznań region of Greater Poland (68 ± 12 years old; 45.0% women; *n* = 260) admitted for treatment at the Department of Neurology, Poznań University of Medical Sciences Hospital, and enrolled from June 2017 to March 2019. Characteristics of ischemic stroke patients are shown in Table S4. Stroke was confirmed by using the criteria of the National Survey of Stroke [31], which requires a set of neurological deficits lasting ≥24 h. Exclusion criteria were (i) hemorrhagic stroke; (ii) uncertain or other causes of stroke; (iii) malignant tumor, surgery, AMI, or trauma within previous three months; (iv) gastrointestinal, autoimmune, inflammatory, thyroid gland, diabetes, renal, or liver disease. Healthy control participants (50 ± 17 years old; 59.7% women; *n* = 291) were enrolled in the study at the same time. Exclusion criteria for healthy control participants were (v) an ischemic vascular event within the previous 12 months and (vi) other diseases. In total, 9 control individuals who had an ischemic stroke after enrollment were excluded, leaving 291 participants in the control group. Participants were eligible for inclusion if they were ≥18 years old. This sample size has ≥0.8 power to detect a 1.15-fold change in the analyzed variables between the groups, based on SD = 50% and a two-sided *p* < 0.05. Participant characteristics and blood/urine samples were collected upon admission to the study. Serum, citrated plasma, and EDTA plasma were prepared as previously described [32]. Ischemic stroke patients had earlier coronary stenosis (23.3%) (>50% of cross-surface area obstructed in at least one of the major coronary arteries), AMI (8.7%), other heart disease (22.5%), diabetes (24.0%), and hypertension (77.5%). The frequency of these risk factors was up to 10 times less prevalent in control individuals: 2.3%, 0.7%, 4.3%, 3.7%, and 21.2%, respectively. Stroke patients were on medications at admission (78.3%), including antiplatelet drugs (11%), acetylsalicylic acid (30%), statins (30%), β-blockers (41%), and metformin (19%). Medication use in control participants was 7.7%, including antihypertensives (3%), statins/anti-lipid (2%), β-blockers (1.4%), and metformin (0.3%). Samples were assayed by investigators blinded to the clinical data to avoid bias. Written informed consent was obtained upon admission to the study. The study protocols followed the principles of the Declaration of Helsinki and were approved by the Bioethical Committee, Poznań University of Medical Sciences (#908/17, #909/17, approved 7 September 2017; #1240/17, approved 15 December 2017).

### 2.2. The Clotting/Lysis Assay

The assay was changed from what has been described previously [12]. Briefly, 25 µL citrated plasma was added to 75 µL buffer (50 mM Tris-HCl, 150 mM NaCl, pH 7.6) containing 12.5 ng tPA (Molecular Innovations), with an 83 ng/mL final concentration. Reactions were started by adding 50 µL of activation mix (0.09 U/mL thrombin (Millipore-Sigma, St. Louis, MO, USA), 22.5 mM CaCl_2_, 50 mM Tris-HCl, 150 mM NaCl, pH 7.6) to each well of a 96-well plate using a multichannel pipette at 20 s intervals. Absorbance was read at 340 nm every 30 s for 1 h using a NanoQuant Infinite M200 Pro microplate reader (Tecan, Männedorf, Switzerland). Complete lysis of fibrin clots occurred within 1 h at the tPA concentration used. Plasma samples were assayed in duplicates, and the values were averaged.

### 2.3. Clotting/Lysis Data Analysis

Kinetics of fibrin clot formation and lysis, illustrated in Figure S1, were analyzed using customized software kindly provided by Dr. Peter Grant [12]. Maximum absorbance at 340 nm (Abs_max_, a measure of fibrin network density) and fibrin CLT (i.e., the time at which A_340_ was reduced to 50% of the highest value, a measure clot’s susceptibility to lysis) were calculated from the kinetics. Inter-assay variabilities for Abs_max_ and fibrin CLT were 1.5% and 4.7%, respectively. Correlations between the clotting and lysis variables for the stroke patients and the healthy individuals are shown in Table S3.

### 2.4. APOE Genotyping

APOE T>C C112R rs429358 was genotyped with the use of TaqMan probes (Thermo Fisher Scientific, Gliwice, Poland, Assay ID C_3084793_20).

### 2.5. Metabolite Assays

Plasma metabolites were quantified by standard assays [32].

#### Metallic and Nonmetallic Element Quantification

We used inductively coupled plasma mass spectrometry (ICP-MS) and inductively coupled plasma optical emission spectrometry (ICP-OES) to quantify serum Li, Ni, Be, Sr, B, S, and Si, which are not known to be associated with fibrin clot properties and ischemic stroke. We also quantified Fe, Cu, Zn, Ca, Sr, Mg, Na, K, Al, and P, which have been previously reported to be associated with ischemic stroke but are not known to be associated with fibrin clot properties. Serum samples (100 μL) were mineralized with a mixture of redistilled nitric acid (70%, 300 μL), hydrogen peroxide (25–35% for ultra-trace analysis, 100 μL), and hydrochloric acid (30% suprapure, 100 μL) for 24 h. Serial dilutions of ICP-MS single-element standard solutions were used for calibration. Additionally, for ICP-MS, Sc, Rh, and Ge ≥ 99.999% purity dissolved in 1% HNO_3_ were used as internal online standards (automatically added during analysis through T-piece). Reagents and standards for mineralization were from Merck, Poland. Deionized water was obtained from the Milli-Q Direct 8 Water Purification System (Merck Millipore). Standard Reference Material (SRM) 909c was used for serum Fe measurements. The certified reference materials BCR 637 (Institute for Reference Materials and Measurements) and ERM-DA120 (European Reference Materials) were analyzed to confirm the calibration for Cu and Al, respectively. Analyses agreed with the certified values, with recovery rates from 93% to 104%.

Analyses were carried out using ICP-MS and ICP-OES instruments, models 2030 and 9820, respectively (Shimadzu, Tokyo, Japan). Detailed information about the instrumentation and validation is shown in Tables S1 and S2. The intra-assay variability values, determined with six assays of the same sample in one run, were 1.3% and 6.4% for Cu and Fe, respectively. The inter-assay variability values, determined with 20 samples assayed on different days, were 1.7%, 13.2%, 20.1%, and 2.1% for Cu, Fe, Al, and Si, respectively. Accuracy and precision for other elements were <3%. Element detection rates were 100% for Fe, Cu, Zn, Ni, Ca, Sr, Mg, Li, Na, K, Al, P, S, and Si. Detection rates for Al and Be were 91.8% and 42.4%, respectively. Values below the limit of detection were replaced by the detection limit divided by the square root of 2 [33].

### 2.6. Statistics

Normality of distribution was tested with Shapiro–Wilk’s statistic. Mean ± standard deviation and median were calculated for normally and non-normally distributed variables, respectively. An unpaired two-sided *t*-test was used for comparisons between two groups of variables with normal distribution. A Mann–Whitney rank sum test was used for comparisons between two groups of non-normally distributed variables. Associations between fibrin CLT, Abs_max_, and elements were studied by bivariate correlations. Possible confounding by other variables (age, sex, traditional risk factors, earlier disease, and medication use) was examined in multiple regression models.

Associations between ischemic stroke, metallic/nonmetallic elements, fibrin CLT, and Abs_max_ were studied by logistic regression in models adjusted for possible confounding variables. Model 1 was adjusted for age and sex. Model 2 was additionally adjusted for glucose, LDL-C, HDL-C, triglycerides, *ApoE112* genotype, GFR, and earlier disease (CAD, MI, hypertension, diabetes, other heart disease, and medication use). To find possible interactions between metallic and nonmetallic elements, we examined models without metallic elements (Model 4) and without metallic or nonmetallic elements (Model 3). Interactions between individual metallic elements were examined in Model 1 (without Li, Na, Ca, and Al), Model 2 (without Na, Ca, and Al), Model 3 (without Li, Ca, and Al), Model 4 (without Li, Na, and Al), and Model 5 (without Li, Na, and Ca). Observations that had missing values were ignored. The statistical software packages Statistica, version 13 (TIBCO Software Inc., Palo Alto, CA, USA), and PSPP, version 1.0.1 (www.gnu.org) (accessed on 15 January 2024), were used. Probability values were 2-sided, and a *p*-value < 0.05 was considered significant.

## 3. Results

The study participants included 260 ischemic stroke patients, 68 years old, and 45.0% were women. Healthy controls included 291 participants, 50 years old, and 59.7% were women. Descriptive statistics of variables analyzed in the present study are shown in Table S4.

### 3.1. Levels of Metallic and Nonmetallic Elements in Ischemic Stroke Patients and Healthy Controls

Levels of plasma Fe, Zn, Ni, Ca, Sr, Mg, and Na were significantly reduced in female and male stroke patients compared with healthy controls (Table 1). However, changes in levels of other metallic elements in stroke patients were sex-specific. For example, Cu, Li, and Al levels were significantly reduced in female stroke patients but unaffected in male stroke patients compared with healthy controls (Table 1). By contrast, K levels were significantly lowered, while beryllium levels were significantly elevated in male stroke patients but unaffected in female stroke patients compared with healthy controls (Table 1).

As expected, serum creatinine was significantly elevated and GFR was reduced in female and male stroke patients compared with healthy controls (Table 1).

### 3.2. Stroke Status Affects Correlations between Serum Elements

Numerous correlations between serum elements were found in the healthy controls. However, some of these correlations were lost in stroke patients. For instance, Ni was significantly associated with Cu, Ca, K, and Al in healthy controls but not in stroke patients. Zn was associated with Ni, Cu, Ca, Sr, Mg, Li, Na, Be, Al, boron (B), and sulfur (S) but not in stroke patients (Table S5). Beryllium was associated with Cu, Zn, Ca, Sr, and K in healthy controls but not in stroke patients. In addition, new correlations, absent in healthy individuals, appeared in stroke patients. For example, Na was correlated with K and Si; K and Sr were correlated with Si; and Fe was correlated with Ni, Zn, Na, K, Be, Al, B, phosphorus (P), and S only in stroke patients (Table S5). Other correlations—e.g., Ca with Cu, Na, K, Be, P, and S; Fe with Si; Be with Al, B, and Si—were seen both in healthy controls and stroke patients (Table S5). These findings suggest fundamental changes in metallic and nonmetallic element homeostasis in stroke patients.

### 3.3. Fibrin Clot Properties in Ischemic Stroke Patients and Healthy Controls

Sex significantly affected CLT in stroke patients (longer in females vs. males; *p* = 0.001) but not in healthy individuals (*p* = 0.199) (Table 1). Fibrin CLT was significantly longer in female (*p* = 0.000) but not male (*p* = 0.181) stroke patients compared with healthy controls (Table 1). However, sex did not affect fibrin Abs_max_ in stroke patients: Abs_max_ was significantly elevated both in female (*p* = 0.000) and male (*p* = 0.003) stroke patients compared with healthy controls (Table 1).

**Figure 1:**
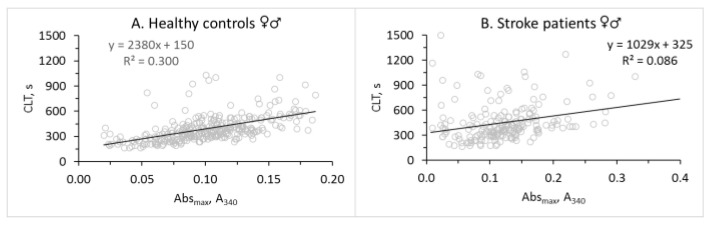

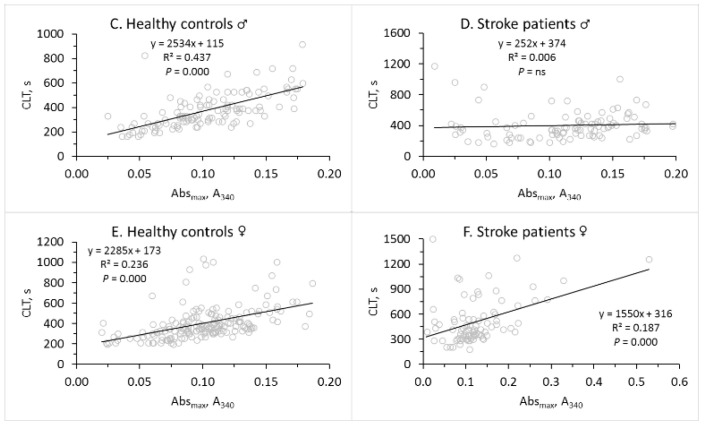
Relationships between fibrin CLT and Abs_max_ in healthy controls (**A**,**C**,**E**) and ischemic stroke patients (**B**,**D**,**F**). Relationships for total (**A**,**B**) and stratified by sex cohorts are shown: male (**C**,**D**); female (**E**,**F**).

A relatively strong fibrin CLT vs. Abs_max_ association in healthy controls (R^2^ = 0.300, *p* = 0.000; Figure 1A) was attenuated in stroke patients (R^2^ = 0.086, *p* = 0.000; Figure 1B). This was caused by a loss of CLT vs. Abs_max_ association in male stroke patients (R^2^ = 0.008, *p* = ns; Figure 1D); CLT and Abs_max_ remained associated in female stroke patients (R^2^ = 0.187, *p* = 0.000; Figure 1F). In healthy males and females, CLT and Abs_max_ were strongly associated (males: R^2^ = 0.437, *p* = 0.000; Figure 1C—females: R^2^ = 0.236, *p* = 0.000; Figure 1E).

We also investigated the relationships of metallic and nonmetallic elements with fibrin clot properties in healthy individuals and ischemic stroke patients.

**Figure 2:**
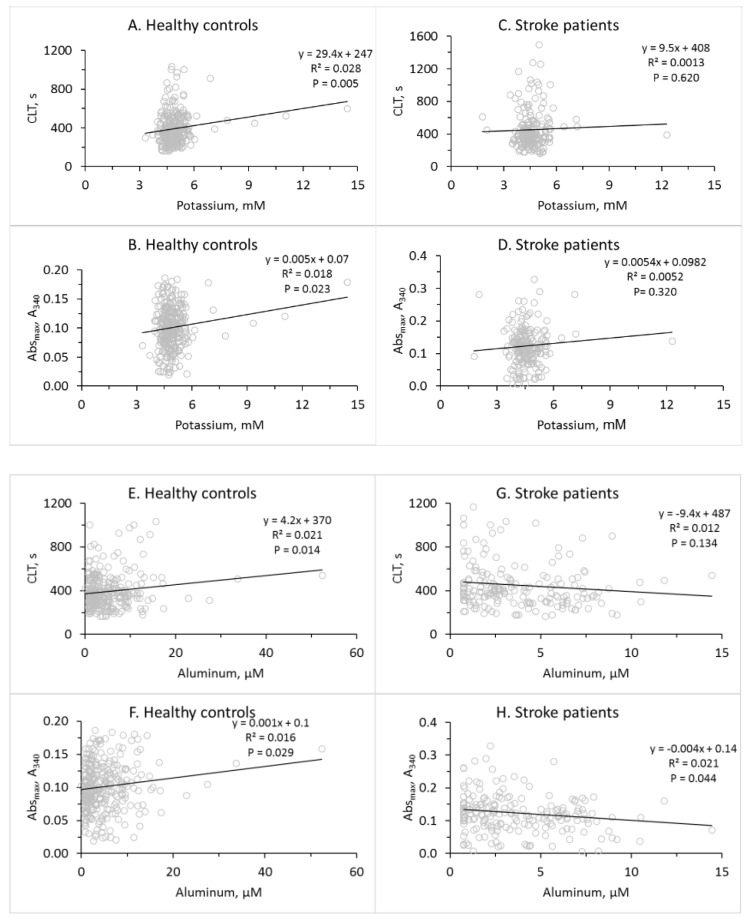

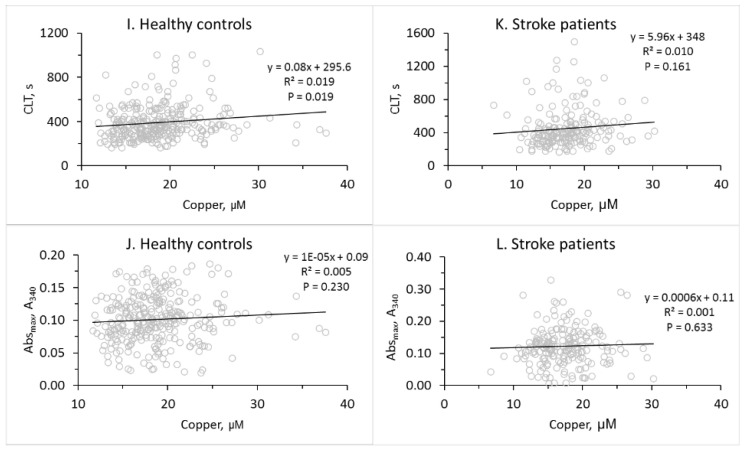
Pearsons’s correlations between plasma metallic elements and fibrin clot properties in healthy controls and stroke patients. Potassium (**A**–**D**), aluminum (**E**–**H**), and copper (**I**–**L**). CLT (**A**,**C**,**E**,**G**,**I**,**K**) and Abs_max_ (**B**,**D**,**F**,**H**,**J**,**L**). Healthy controls (**A**,**B**,**E**,**F**,**I**,**J**) and stroke patients (**C**,**D**,**G**,**H**,**K**,**L**).

In healthy individuals, potassium was significantly associated with fibrin CLT (Figure 2A) and Abs_max_ (Figure 2B). Aluminum was also significantly associated with CLT (Figure 2E) and Abs_max_ (Figure 2F), while copper was associated only with fibrin CLT (Figure 2I) but not with fibrin Abs_max_ (Figure 2J) (Table 2).

In contrast, in ischemic stroke patients, potassium was not associated with fibrin CLT (Figure 2C) or Abs_max_ (Figure 2D). There was also no association of aluminum (Figure 2G) and copper (Figure 2K) with fibrin CLT and no association of copper with fibrin Abs_max_ (Figure 2L). However, there was a significant negative association between aluminum and fibrin Abs_max_ in ischemic stroke patients (Figure 2H) (Table 2).

In healthy individuals, zinc and calcium were not associated with fibrin CLT (Figures S2A and S2E, respectively) or Abs_max_ (Figures S2B and S2F, respectively). Beryllium was also not associated with fibrin CLT (Figure S2I) or Abs_max_ (not shown). Sulfur was significantly associated with fibrin CLT (Figure S2K) and Abs_max_ (Figure S2L). Other metals (Na, Li, Sr, Fe, Mg, and Ni) and nonmetals (B, Si, and P) were not associated with fibrin CLT or Abs_max_ in healthy controls (Table 2).

In ischemic stroke patients, zinc and calcium became associated with Abs_max_ (Figure S2D,H) but not with CLT (Figure S2C,G), while beryllium became significantly associated with CLT (Figure S2J) but not with Abs_max_ (not shown). Sulfur was significantly associated with fibrin Abs_max_ (R^2^ = 0.049, *p* = 0.000; Figure S2N) but not with CLT (Figure S2M). As in healthy controls, other metals (Na, Li, Sr, Fe, Mg, and Ni) and nonmetals (B, Si, and P) were not associated with fibrin CLT or Abs_max_ in stroke patients (Table 2).

These findings suggest that higher levels of serum K, Al, and Cu in healthy individuals promote a fibrin clot structure that is more compact and less susceptible to lysis. However, these associations between K, Al, and Cu and fibrin clot properties were not seen in stroke patients in whom Zn, Ca, Be, and S became associated with fibrin clot properties.

We also examined the relationships of metals and silicon with age and GFR in ischemic stroke patients and healthy individuals.

### 3.4. Age

In healthy individuals, serum sodium (Figure S3A), lithium (Figure S3C), copper (Figure S3E), beryllium (Figure S3I), and aluminum (Figure S3K) significantly increased, while iron (Figure S3G) and silicon (Figure S3M) decreased with age. Strontium (Figure S3O) and other metals (K, Zn, Ni, Mg, and Ca; Table 2) were not affected by age in healthy individuals.

By contrast, in ischemic stroke patients, age was not associated with sodium (Figure S3B), lithium (Figure S3D), copper (Figure S3F), iron (Figure S3H), beryllium (Figure S3J), or aluminum (Figure S3L), while silicon significantly increased with age (*p* = 0.015; Figure S3N). Strontium, which was not affected by age in healthy individuals (Figure S3O), significantly increased with age in stroke patients (*p* = 0.035; Figure S3P). Metals that were not affected by age in healthy individuals (K, Zn, Ni, Mg, and Ca), were also not affected by age in stroke patients (Table 2).

These findings suggest that stroke affects the homeostasis of metallic/nonmetallic elements by abrogating the positive associations of sodium, lithium, copper, iron, beryllium, and aluminum with age seen in healthy control individuals. The associations of age with silicon and strontium became positive in stroke patients.

### 3.5. GFR

In healthy individuals, GFR was significantly negatively associated with sodium (Figure S4A), potassium (Figure S4C), lithium (Figure S4E), strontium (Figure S4G), copper (Figure S4I), and aluminum (Figure S4K). Other metals (Be, Fe, Ca, Mg, Zn, and Ni; Table 2) and silicon (Figure S4M) were not associated with GFR in healthy individuals.

By contrast, in ischemic stroke patients, GFR was not associated with sodium (Figure S4B), potassium (Figure S4D), lithium (Figure S4F), or aluminum (Figure S4L). Strontium and copper, which were negatively associated with GFR in healthy individuals (Figures S4G and S4I, respectively), were also negatively associated with GFR in stroke patients (Figures S4H and S4J, respectively). Silicon, which was not associated with GFR in healthy individuals (Figure S4M), became significantly negatively associated with GFR in stroke patients (Figure S4N). Metallic elements (Be, Fe, Ca, Mg, Zn, and Ni) that were not associated with GFR in healthy individuals were also not associated with GFR in stroke patients (Table 2).

These findings suggest that stroke affects the homeostasis of metallic/nonmetallic elements by abrogating the negative associations of sodium, potassium, lithium, and aluminum with GFR seen in healthy control individuals. The association of GFR with silicon became positive in stroke patients.

### 3.6. Determinants of Fibrin CLT and Abs_max_ in Stroke Patients and Healthy Individuals

#### 3.6.1. Bivariate Correlations

In bivariate correlation analysis in healthy individuals, fibrin CLT was significantly associated with three metallic elements (K, Cu, and Al) and eight other variables: total cholesterol, LDL-C, triglycerides, BMI, hypertension, earlier CVD, age, and fibrin Abs_max_ (Table 3).

In stroke patients, fibrin CLT was associated with a different set of variables that included one metallic element (Be) and four other variables: glucose, sex, earlier CVD, and Abs_max_ (Table 3).

In bivariate correlation analysis, fibrin Abs_max_ was associated with different sets of variables. In healthy individuals, fibrin Abs_max_ was associated with two metallic elements (K, Al) and eight other variables: total cholesterol, LDL-cholesterol, triglycerides, BMI, hypertension, other heart disease, age, and fibrin CLT (Table 3).

In stroke patients, fibrin Abs_max_ was associated with a different set of variables that included three metallic elements (Zn, Ca, and Al) and fibrin CLT (Table 3).

#### 3.6.2. Multiple Regression Analysis

In multiple regression analysis, fibrin CLT and Abs_max_ were associated with different sets of variables (Table 3). For example, in healthy individuals, CLT was positively associated with three metallic elements (K, Be, and Al) and two other variables: sex and Abs_max_; the adjusted R^2^ was 0.32, and *p* = 0.000 (Table 3).

In stroke patients, fibrin CLT was associated with a different set of variables that included two metallic elements (Ca; Be) and five other variables: glucose, total cholesterol, GFR, sex, and Abs_max_; the adjusted R^2^ was 0.21, and *p* = 0.000 (Table 3).

In a multiple regression analysis of healthy individuals, fibrin Abs_max_ was associated with one metallic element (Na) and four other variables: BMI, age, sex, and CLT; the adjusted R^2^ was 0.38, and *p* = 0.000 (Table 3).

In stroke patients, Abs_max_ was associated with a different set of variables that included five serum elements (Zn, Ca, K, Be, and Si) and two other variables: fibrin CLT and triglycerides; the adjusted R^2^ was 0.27, and *p* = 0.000 (Table 3).

Overall, metals explained a greater part of the variability in Abs_max_ in stroke patients (the adjusted R^2^ was 0.27 in the presence of metals and 0.09 in the absence of metals) than in the healthy controls (the adjusted R^2^ was 0.38 in the presence of metals and 0.36 in the absence of metals) (Table 3). A smaller part of the variability in CLT was explained by metals in stroke patients (the adjusted R^2^ was 0.21 in the presence of metals and 0.18 in the absence of metals) and healthy controls (the adjusted R^2^ was 0.32 in the presence of metals and 0.30 in the absence of metals). In stroke patients, silicon (Si) explained only a small fraction of the variability in Abs_max_ (the adjusted R^2^ was 0.09 in a model without metals and 0.08 in a model without Si and metals) but none in CLT (Table 3).

These findings show that five metallic elements contribute to the variability in fibrin Abs_max_ and CLT in stroke patients (Zn, Ca, K, B, and Al) but only one in healthy individuals (Na). Silicon contributes to Abs_max_ variability only marginally in stroke patients.

### 3.7. Associations of Metallic/Nonmetallic Elements and Fibrin Clot Properties with Ischemic Stroke

#### 3.7.1. Bivariate Correlations

In bivariate correlation analysis, twelve metallic (Fe, Cu, Zn, Ni, Ca, Sr, Mg, Li, Na, K, Be, and Al) and two nonmetallic elements (Si; P) were associated with ischemic stroke, as were fibrin CLT and Abs_max_ (Table 4). Variables such as glucose; lipid measures; GFR; age; sex; and earlier CAD, MI, hypertension, diabetes, and other heart diseases were also associated with ischemic stroke in bivariate analyses (Table 4).

#### 3.7.2. Logistic Regression

In a logistic regression analysis adjusted for age and sex (Model 1, Table 4), five metallic (Na, Li, Ca, Sr, and Ni) and two nonmetallic elements (Si; B) were significantly associated with ischemic stroke, while other metallic (Fe, Cu, Zn, Mg, K, Be, and Al) and nonmetallic elements (P; S) were not.

After other adjustments for traditional risk factors such as glucose, GFR, LDL-C, HDL-C, triglycerides, *APOE112* polymorphism, earlier disease, and medications (Model 2, Table 4), four metallic (Na, Ca, Sr, and Ni) and one nonmetallic element (Si) remained significantly associated with ischemic stroke, while three more metallic elements (K, Be, and Al) became significant.

As also shown in Table 4, fibrin CLT and Abs_max_ were not associated with ischemic stroke in Model 1, adjusted for age and sex, or in a fully adjusted Model 2. However, fibrin Abs_max_ was associated with stroke in a model without metallic and nonmetallic elements (Model 3) or only without metallic elements (Model 4). This showed that metallic but not nonmetallic elements abolished the association of fibrin Abs_max_ with ischemic stroke.

To figure out which metallic element can abolish the association of Abs_max_ with ischemic stroke, we examined the effects of individual metals on this association. As shown in Table 5, we found that Abs_max_ was associated with ischemic stroke in a model with eight metallic elements (K, Sr, Ni, Be, Mg, Fe, Cu, and Zn; Model 5). By contrast, this association was abolished in models in which just four metallic elements (Li, Na, Ca, and Al) were present together (Model 5) or separately: Li in Model 6, Na in Model 7, Ca in Model 8, or Al in Model 9 (Table 5).

Although Fe did not affect the association between Abs_max_ and ischemic stroke and was associated with neither (Table 3; Model 5, Table 5), it became significantly associated with ischemic stroke in models without Ca or Al (Models 6, 7, and 8). These findings suggest that Fe interacts with Ca and Al. The association of potassium (K) with ischemic stroke (Model 5, Table 5) was lost in models without Li, Ca, Be, or Al (Model 8) and Li, Na, or Al (Model 9), suggesting an interaction between K and Li, Ca, Be, Na, or Al. Boron (B) was associated with ischemic stroke in models with Na (Models 5 and 8) but not in models without Na, suggesting an interaction between B and Na. Although sulfur (S) did not affect the association of Abs_max_ with ischemic stroke and was associated with neither (Table 3; Model 5, Table 5), it became significantly associated with ischemic stroke in a model without Li, Na, and Al (Model 9, Table 5). These findings suggest interactions between S and Li, Na, or Al.

#### 3.7.3. Contribution of Individual Elements to the Risk of Ischemic Stroke

To estimate the contributions of individual elements to ischemic stroke risk, we examined how each of these elements affects R^2^ values in the logistic regression models described in Table 4. We found that all metals explained 16% of ischemic stroke risk in an unadjusted model, with the highest contributions from Na (7%) and Si (1.5%). Other elements explained <1 to 1% of the risk (Model 1, Table 6). In the adjusted model, all metals explained 13.5% of ischemic stroke risk, with the highest contributions from Na (4%) and Si (1.5%), while other elements explained <1 to 1% of the risk (Model 2, Table 6).

## 4. Discussion

We found that (i) different sets of elements were associated with fibrin clot properties (CLT and/or Abs_max_) in stroke patients (zinc, calcium, potassium, beryllium, and silicon) and healthy controls (sodium, potassium, beryllium, and aluminum); (ii) serum lithium, sodium, potassium, calcium, strontium, beryllium, nickel, aluminum, silicon, and boron were associated with ischemic stroke while zinc was not; (iii) iron, copper, magnesium, sulfur, and phosphorus were neither associated with fibrin clot properties nor stroke; (iv) fibrin clot properties were associated with stroke in models with nonmetals but not in models with metals; (v) lithium, sodium, calcium, and aluminum abrogated the association of fibrin clot properties with ischemic stroke, while nickel, potassium, strontium, and beryllium did not; (vi) interactions between metallic/nonmetallic elements changed their associations with ischemic stroke.

In addition to stroke-associated changes in the levels of metallic and nonmetallic elements, we found profound changes in the relationships of these elements with fibrin clot properties, age, and GFR in stroke patients (Table 2). For example, the positive associations of fibrin clot properties (CLT, Abs_max_) with potassium, aluminum, and copper (Figure 2) and the negative association of CLT with sulfur (Figure S2K,L) observed in healthy individuals were lost in stroke patients (Figure 2, Figure S2M). Further, the significant negative associations of fibrin Abs_max_ with zinc and calcium and fibrin CLT with beryllium found in stroke patients were not seen in healthy controls (Figure S2).

The positive associations between age and serum sodium, lithium, copper, beryllium, and aluminum observed in healthy individuals were lost in stroke patients (Figure S3). The negative association between serum iron and age observed in healthy individuals (Figure S3K) was also lost in stroke patients (Figure S3L). In contrast, serum strontium, which was not associated with age in healthy controls (Figure S3O) became positively associated with age in stroke patients (Figure S3P). Serum silicon was also positively associated with age in stroke patients (Figure S3N) but negatively in healthy controls (Figure S3M).

Negative associations between GFR and serum sodium, potassium, lithium, and aluminum in healthy individuals were not seen in stroke patients (Figure S4A–L). However, negative associations between serum strontium and GFR were seen both in healthy controls (Figure S4G) and stroke patients (Figure S4H). Notably, a negative association between silicon and GFR was seen in stroke patients (Figure S4N) but not in healthy individuals (Figure S4M). These findings suggest profound changes in metal and silicon homeostasis associated with ischemic stroke. Whether these changes were causally related to stroke is still to be figured out in future studies.

Notably, we found that the CLT vs. Abs_max_ correlation was sex-dependent in stroke patients but not in healthy controls. Specifically, the positive correlation between CLT and Abs_max_ observed in healthy males (Figure 1C) was absent in male stroke patients (Figure 1D). However, the positive correlation between CLT and Abs_max_ observed in healthy females (Figure 1E) was also seen in female stroke patients (Figure 1F). As studies of other diseases (diabetes [34,35], CAD [32], and MI [36]) have not analyzed fibrin clot properties separately in women and men, it is not known whether the sex-specific effect on the CLT vs. Abs_max_ correlation is unique to ischemic stroke or is characteristic of other diseases as well.

To the best of our knowledge, associations between metallic/nonmetallic elements and fibrin clot properties in relation to ischemic stroke have not been reported before. In the present study, multivariate regression analysis showed that metallic and nonmetallic elements were associated with fibrin clot properties in stroke patients and healthy controls. Specifically, in healthy individuals, metallic elements such as potassium, beryllium, and aluminum were significantly associated with CLT, while sodium was associated with Abs_max_ (Table 3). A different set of metallic elements and silicon were associated with fibrin clot properties in ischemic stroke patients: calcium and beryllium were significantly associated with fibrin CLT and Abs_max_, while zinc, potassium, and aluminum were associated with fibrin Abs_max_ (Table 3).

Logistic regression analyses showed that metallic (lithium, sodium, potassium, calcium, strontium, nickel, beryllium, and aluminum) and nonmetallic (silicon; boron) elements were associated with ischemic stroke (Table 4). These associations were independent of other elements and traditional risk factors such as age, sex, glucose, LDL-C, HDL-C, triglycerides, GFR, earlier diseases, and medication use. To the best of our knowledge, associations between ischemic stroke and serum lithium, strontium, nickel, beryllium, silicon, and boron, supported by logistic regression analyses, have not been reported before.

Interestingly, we found that the associations of some elements with ischemic stroke were affected by other elements. For example, iron (Fe) was significantly associated with ischemic stroke in models without calcium (Ca) or aluminum (Al) (Models 6, 7, and 8) but not in a final logistic regression model (Model 5, Table 5). Potassium (K) was associated with ischemic stroke in Model 5 but not in models without lithium (Li), Ca, beryllium (Be), or Al (Model 8) or Li, sodium (Na), or Al (Model 9). Boron (B) was associated with ischemic stroke in models with Na (Models 5 and 8) but not in models without Na. Sulfur (S) was significantly associated with ischemic stroke in a model without Li, Na, or Al (Model 9, Table 5) but not in a final logistic regression model (Model 5, Table 5). These findings suggest interactions of Fe with Ca and Al; K with Li, Ca, Be, Al, and Na; B with Na; and S with Li, Na, and Al. Some of these interactions were also seen in binary correlations of Fe with Al; K with Ca and Na in ischemic stroke patients but not in healthy controls (Table S5).

A small study reported significantly elevated serum lithium, nickel, and boron and no changes in aluminum and strontium levels in male ischemic stroke patients vs. control males [30]. However, in the present work, we found that Li, Ni, B, and Al were lowered while Sr was elevated in ischemic stroke patients vs. healthy controls (Table 1). These differences might be due to different ethnicities (Poznań vs. Moscow) and/or the sizes of the studied populations (260 cases and 291 controls (Table 1) vs. 30 cases and 30 controls [30]). The associations of Al, Na, K, and Ca with stroke in the Polish Central European population of the Poznań region of Greater Poland (Table 4) are consistent with previous extensive studies in other populations [23,37,38,39].

The associations of serum Li, Sr, Ni, Be, and Si with ischemic stroke found in the present study are biologically plausible. Lithium, which has been used for over 100 years to treat manic depression, has many effects on the brain and blood [40]. It is neuroprotective in animal models of stroke, inhibiting the activation of nucleotide-binding oligomerization domain-like receptor family pyrin domain-containing 3 (NLRP3) inflammasomes in a middle cerebral artery occlusion (MCAO) stroke model in mice. This effect of lithium is mediated by AKT/GSK3β/β-catenin and AKT/FoxO3a/β-catenin signaling pathways, which suppress the production of reactive oxygen species [41]. Lithium, an inhibitor of GSK-3β, activates mTOR through the Wnt/GSK-3β pathway, inhibits autophagy, and improves ischemic brain damage in the MCAO rat model of stroke [42]. However, excess lithium can cause intoxication with neurological symptoms imitating stroke [43].

Strontium levels in drinking water and urine are negatively correlated with cardiovascular mortality, showing that Sr can have a protective effect on the vasculature [44]. Sr can compete with sodium for transport in the intestinal lumen, promoting Na excretion [44]. In a bone regeneration study, Sr promoted angiogenesis [45]. However, excess Sr can adversely affect the cardiovascular system. For example, excess plasma Sr can accumulate in aortic valve plaques and contribute to the calcification of blood vessels [46].

Nickel is an essential metal that can inhibit cytosine 5-methyltransferase activity in vivo and in vitro and elevate genomic DNA methylation in Chinese hamster cells even when DNA methyltransferase activity is depressed [47]. Nickel can induce mitochondrial damage [48] and autophagy through the Akt and APMK/mTOR pathways [49] in mouse kidney cells in vitro and in vivo. Treatments with nickel induced oxidative stress, DNA damage, and apoptosis in rat testes [50,51]. Nickel exposure can also inhibit the antioxidant enzyme superoxide dismutase, produce reactive oxygen species, and induce lipid peroxidation and oxidative DNA damage (reviewed in [52]).

Beryllium is a toxic metal that is linked to lung diseases, including pulmonary heart disease. Be toxicity is underlined by its ability to induce ferroptosis and ferritinophagy in epithelial cells. Interestingly, these processes can be alleviated by treatments with hydrogen sulfide, which suggests a possible strategy for the prevention and treatment of beryllium toxicity [53].

Silicon, one of the elements essential for human health, has been linked to brain function by affecting some aspects of cognition. Specifically, in multiple regression analyses in patients with mild cognitive impairment, silicon has been found to be associated with cognition in the attention/processing speed domain [54]. Si activates Wnt5a/Ca^+2^ signaling and endoplasmic reticulum stress, leading to mitochondrial redox imbalance and ferroptosis in mouse macrophages, a process that underlies silicosis [55]. Si is also beneficial for bone health [56] and acts synergistically with Sr on osteogenesis, osteoclastogenesis, and angiogenesis in osteoporotic bone regeneration [45]. Supplementation with silicon is a beneficial treatment for the photodamaged skin [57].

Notably, we found that a set of elements associated with stroke was different from those associated with fibrin clot properties. Some of the elements that were associated with stroke (Na, K, Ca, Be, Al, and Si) (Table 4) were also associated with fibrin clot properties (Table 3). However, other elements associated with stroke (Li, Sr, Ni, and B) were not associated with fibrin clot properties. Zinc, which was not associated with stroke (Table 4), was nevertheless associated with fibrin Abs_max_ (Table 3). These findings suggest that (a) the association of Na, K, Ca, Be, Al, and Si with stroke reflects the possible influence of these elements on fibrin clot properties; (b) Li, Sr, Ni, and B may promote stroke without influencing fibrin clot properties; and (c) some elements, such as zinc, which can influence fibrin clot properties, do not appear to influence stroke. However, the suggestion that the association of Na, K, Ca, Be, Al, and Si with stroke reflects the possible influence of these elements on fibrin clot properties is not tenable because fibrin clot properties were not associated with ischemic stroke in logistic regression models that included Li, Na, Ca, or Al (Table 4 and Table 5).

Our finding that fibrin clot properties were not associated with ischemic stroke (Table 4 and Table 5) seemed to be at odds with earlier studies of relationships between ischemic stroke and fibrin clot properties [9,10,11]. One study reported that CLT and Abs_max_ were significantly elevated in 147 stroke patients compared with 120 healthy controls [9]. A second study also reported that CLT and Abs_max_ were significantly elevated in 45 stroke patients compared with 45 healthy controls and that stroke was a significant predictor of Abs_max_ but not CLT in linear regression models adjusted for age, sex, fibrinogen, glucose, creatinine, Lp (a) levels, and stroke [10]. The conclusions of a third study about the association between CLT and ischemic stroke were based on logistic regression models adjusted for traditional risk factors [11]. However, the major difference between the present study and earlier studies is the inclusion of metallic elements in the present study, which accounts for the different conclusions. Indeed, we found that fibrin Abs_max_ but not CLT was associated with ischemic stroke in logistic regression models without metallic elements (Models 3 and 4, Table 4). We also found that lithium, sodium, calcium, and aluminum abolished the association of Abs_max_ with ischemic stroke, while other metals (nickel, potassium, strontium, beryllium, magnesium, iron, copper, and zinc) and nonmetals (boron, silicon, phosphorus, and sulfur) did not (Table 5).

Fibrin clot properties have been reported to be associated with CVD in case–control and prospective studies. For example, a case–control study with 800 AMI patients and 1123 controls found that long CLT and high Abs_max_ were associated with an increased risk of AMI [36]. A case–control study that reported the association of CLT with ischemic stroke (175 cases; 638 controls) also reported that CLT was associated with AMI (203 cases; 638 controls) [11]. A prospective study of 300 patients hospitalized with acute coronary syndrome found that a long CLT was associated with an increased risk of death, nonfatal AMI, and stroke at a 12-month follow-up [58]. CLT and Abs_max_ predicted AMI and mortality in a large PLATO study involving 4354 patients with acute coronary syndrome [34] and 974 patients with diabetes [35]. Another prospective study of 1952 CAD patients found that a longer CLT and higher Abs_max_ predicted AMI and mortality during a 7-year follow-up [32].

Because risk factors for CVD are like those for stroke [1], it is likely that the association of metals with CVD reported in earlier studies [14,15,16,17,18,19,20] may not be mediated by their effects on fibrin clot properties, as we have shown for ischemic stroke in the present study (Table 4 and Table 5). We predict that the association between fibrin clot properties and AMI seen in several studies will be abolished by the inclusion of metals in regression models. This, however, is still to be investigated in future studies.

To understand the mechanism underlying the association of metals/nonmetals with stroke, further studies of their role in health and disease are warranted. There is also a need to establish reference values for metals/nonmetals in various populations and an assessment of their levels, which may inform us about prevention and treatment strategies. Possible treatments should aim at restoring metal homeostasis, for example, through chelation therapy or Si(OH)_4_ therapy. Another approach would be to ameliorate metal-induced oxidative stress, detrimental changes in mTOR signaling, and autophagy through pharmacological intervention.

Taken together, our present findings suggest that metals and nonmetals, but not fibrin clot properties, can modulate the risk of ischemic stroke. The role of metals and nonmetals in ischemic stroke and their diagnostic value remain to be investigated in future studies.

### Strength and Limitations

The present study is the first to evaluate serum metallic and nonmetallic elements as determinants of fibrin clot properties in relation to ischemic stroke events. To the best of our knowledge, this is also the first study investigating the association between the risk of ischemic stroke and serum lithium, nickel, beryllium, boron, and silicon. The serum levels of twelve metallic and four nonmetallic elements were quantified using ICP-MS ICP-OES instruments, and the data were analyzed using multiple regression and logistic regression models with adjustments for potential confounders. The case–control design of the present study allows for the identification of associations whose mechanistic significance and causality need to be assessed in prospective studies. As the present study was limited to the Polish Caucasian Central European population from the Poznań region of Greater Poland, the generalizability of these findings needs to be examined in other populations in future studies. Even though we have adjusted for potential confounders, we cannot rule out the possibility that diet, occupational exposure, or other lifestyle factors contributed to the associations between metallic/nonmetallic elements, fibrin clot properties, and stroke.

## 5. Conclusions

Using logistic regression analysis, we found novel associations between ischemic stroke and serum Li, Ni, Be, Sr, B, and Si independent of traditional risk factors and confirmed previously the identified associations of ischemic stroke with serum Na, K, Ca, and Al. We also found associations of fibrin clot properties with some of these elements (Na, K, Ca, Be, Al, Si) but not with ischemic stroke. Positive correlations between fibrin clot properties and metals (K, Al, and Cu), as seen in healthy controls, were lost in ischemic stroke patients. These findings show that quantifying metals is important in studies examining relationships between fibrin clot properties and CVD/stroke. Targeting element homeostasis might be a useful therapeutic strategy for mitigating the risk of ischemic stroke.

## Figures and Tables

**Table 1:** Fibrin clot properties and element levels in stroke patients and healthy controls stratified by sex *.

Variable	Stroke Patients (*n* = 260)			Healthy Controls (*n* = 291)			*P*_strokeF_	*P*_strokeM_
	Women	Men	*P*_sex_	Women	Men	*P*_sex_		
	**Fibrin clot properties**							
	(*n* = 85)	(*n* = 106)		(*n* = 173)	(*n* = 118)			
CLT, s	508 ± 264	405 ± 177	**0.001**	401 ± 161	377 ± 177	0.187	**0.000**	0.188
Abs_max_, A_340_	0.124 ± 0.75	0.122 ± 56	0.844	0.100 ± 0.035	0.104 ± 0.037	0.339	**0.000**	**0.003**
	**Metallic elements**							
	(*n* = 151)	(*n* = 109)		(*n* = 173)	(*n* = 118)			
Fe, μM	23.1 ± 14.0	25.1 ± 19.4	0.510	31.4 ± 13.2	36.1 ± 19.4	**0.039**	**0.000**	**0.000**
Cu, μM	18.7 ± 4.0	16.3 ± 3.6	**0.000**	20.3 ± 4.3	16.4 ± 3.6	**0.000**	**0.001**	0.828
Zn, μM	10.7 ± 2.9	11.4 ± 4.0	0.161	11.9 ± 1.6	12.2 ± 4.0	0.171	**0.000**	**0.043**
Ni, μM	0.28 ± 0.19	0.33 ± 0.70	0.524	0.62 ± 0.25	0.63 ± 0.70	0.786	**0.000**	**0.000**
Ca, mM	2.32 ± 0.25	2.27 ± 0.18	0.098	2.53 ± 0.16	2.51 ± 0.18	0.569	**0.000**	**0.000**
Sr, μM	↑0.41 ± 0.29	↑0.36 ± 0.21	0.091	0.31 ± 0.14	0.30 ± 0.21	0.635	**0.000**	**0.000**
Mg, mM	0.83 ± 0.11	0.84 ± 0.09	0.413	0.86 ± 0.06	0.86 ± 0.09	0.696	**0.001**	**0.031**
Li, μM	1.36 ± 1.33	1.43 ± 2.26	0.788	1.78 ± 1.00	1.60 ± 2.26	0.100	**0.003**	0.454
Na, mM	132 ± 20	131 ± 15	0.588	159 ± 9	156 ± 15	**0.010**	**0.000**	**0.000**
K, mM	5.03 ± 1.27	4.40 ± 1.59	**0.001**	4.86 ± 0.47	4.79 ± 1.59	0.412	0.094	**0.019**
Be, μM	0.83 ± 0.23	↑0.84 ± 0.22	0.643	0.77 ± 0.34	0.70 ± 0.22	0.093	0.089	**0.000**
Al, μM	3.98 ± 2.58	4.06 ± 2.70	0.709	5.05 ± 5.38	4.82 ± 2.70	0.711	**0.042**	0.112
	**Nonmetallic elements**							
B, μM	51.0 ± 155.7	28.0 ± 76.1	0.127	27.2 ± 76.8	26.0 ± 76.1	0.896	0.127	0.896
P, mM	3.83 ± 0.89	3.58 ± 0.64	0.006	4.00 ± 0.49	3.64 ± 0.64	0.000	0.065	0.397
S, mM	28.8 ± 4.1	29.0 ± 3.3	0.594	28.6 ± 2.0	29.0 ± 3.3	0.082	0.613	0.967
Si, μM	3.82 ± 1.04	3.66 ± 9.0	0.153	6.04 ± 2.49	5.79 ± 0.90	0.403	**0.000**	**0.000**
	**Other variables**							
Creatinine, μM	↑78 ± 24	↑94 ± 38	**0.000**	64 ± 9	81 ± 38	**0.000**	**0.000**	**0.000**
GFR, mL/min/1.73 m^2^	70.5 ± 18.4	74.5 ± 17.6	0.106	85.6 ± 8.1	87.6 ± 17.6	**0.034**	**0.000**	**0.000**
Age, years	71 ± 12	66 ± 12	**0.000**	53 ± 16	46 ± 12	**0.000**	**0.000**	**0.000**

* CLT, clot lysis time; Abs_max_, maximum absorbance at 335 nm; GFR, glomerular filtration rate. Up arrows, **↑**, indicate variables that were increased in ischemic stroke patients. Significant *P* values are highlighted in **bold**.

**Table 2:** *p*-values for correlations between metallic/nonmetallic elements and age, sex, and GFR in stroke patients and healthy individuals.

Elements	Healthy Controls					Stroke Patients				
	Age	Sex	GFR	CLT	Abs_max_	Age	Sex	GFR	CLT	Abs_max_
	*p* values *									
Na	0.000 (+)	0.010 (−)	0.006 (−)	ns	ns	ns	ns	ns	ns	0.054 (−)
Li	0.000 (+)	ns	0.024 (−)	ns	ns	ns	ns	ns	ns	ns
Cu	0.000 (+)	0.000 (−)	0.049 (−)	0.019 (+)	ns	ns	0.000 (−)	0.039 (−)	ns	ns
Be	0.000 (+)	ns	ns	ns	ns	ns	ns	ns	0.037 (−)	ns
Fe	0.011 (+)	0.038 (+)	ns	ns	ns	ns	ns	ns	ns	ns
Al	0.040 (+)	ns	0.009 (−)	0.014 (+)	0.029 (+)	ns	ns	ns	ns	0.044 (−)
Si	0.003 (−)	ns	0.052 (+)	ns	ns	0.015 (+)	ns	0.009 (−)	ns	ns
Sr	ns	ns	0.001 (−)	ns	ns	0.035 (+)	ns	0.003 (−)	ns	ns
K	ns	ns	0.011 (−)	0.005 (+)	0.023 (+)	ns	ns	ns	ns	ns
Ca	ns	ns	ns	ns	ns	ns	ns	ns	ns	0.002 (−)
Mg	ns	ns	ns	ns	ns	ns	ns	ns	ns	ns
Zn	ns	ns	ns	ns	ns	ns	ns	0.047 (+)	ns	0.007 (−)
Ni	ns	ns	ns	ns	ns	ns	ns	ns	ns	ns
B	ns	ns	ns	ns	ns	ns	ns	ns	ns	ns
P	0.000 (+)	0.000 (−)	0.049 (−)	0.025 (+)	ns	0.014 (−)	0.006(−)	ns	ns	ns
S	0.000 (−)	ns	ns	0.041 (−)	0.000 (−)	0.015 (−)	ns	ns	ns	0.000 (−)

* The signs (−) and (+) indicate negative and positive correlations, respectively. ns, nonsignificant.

**Table 3:** Determinants of plasma fibrin clot lysis time (CLT) and maximal absorbance (Abs_max_) in stroke patients and healthy controls.

	Stroke Patients (*n* = 191)								Healthy Controls (*n* = 291)							
Variable	CLT				Abs_max_				CLT				Abs_max_			
	Pearson Correlation		Multiple Regression *		Pearson Correlation		Multiple Regression *		Pearson Correlation		Multiple Regression *		Pearson Correlation		Multiple Regression *	
	β	*P*	β	*P*	β	*P*	β	*P*	β	*P*	β	*P*	β	*P*	β	*P*
Metals																
Fe		ns			−0.11	0.117										
Cu	0.10	0.161				ns			0.14	0.019			0.07	0.230		
Zn		ns			−0.19	0.007	−0.15	0.025	−0.09	0.115				ns		
Ni		ns				ns				ns				ns		
Ca		ns	0.15	0.044	−0.22	0.002	−0.40	0.000		ns				ns		
Sr		ns				ns				ns				ns		
Mg		ns				ns				ns				ns		
Li		ns				ns				ns				ns		
Na		ns			−0.14	0.054				ns			−0.08	0.157	−0.14	0.003
K		ns				ns	0.23	0.003	0.17	0.005	0.10	0.048	0.13	0.023		
Be	−0.15	0.037	−0.22	0.005		ns	0.39	0.000	−0.09	0.118	−0.13	0.020		ns		
Al	−0.11	0.134			−0.15	0.044	−0.17	0.022	0.14	0.014	0.10	0.046	0.13	0.029		
Nonmetals																
Si		ns	−0.10	0.204	0.12	0.107	0.23	0.003		ns				ns		
B		ns				ns										
P		ns			−0.11	0.134			0.13	0.025			0.10	0.084		
S		ns			−0.26	0.000			−0.12	0.041			−0.22	0.000		
Fibrin clot properties																
Fibrin Abs_max_	0.29	0.000	0.32	0.000					0.55	0.000	0.50	0.000				
Fibrin CLT					0.29	0.000	0.30	0.000					0.55	0.000	0.48	0.000
Other variables																
Glucose	0.16	0.031	0.19	0.006		ns				ns			0.10	0.085		
Total cholesterol	−0.13	0.076	−0.16	0.025		ns			0.18	0.002			0.22	0.000		
LDL−C		ns				ns			0.14	0.013			0.22	0.000		
HDL−C		ns				ns				ns				ns		
Triglycerides		ns			−0.14	0.063	−0.14	0.032	0.11	0.049			0.14	0.013		
GFR		ns	0.17	0.024		ns				ns				ns		
BMI		NA				NA			0.20	0.000			0.31	0.000	0.10	0.048
Earlier CVD	0.16	0.031				ns			0.12	0.040				ns		
Hypertension		ns				ns			0.17	0.003			0.14	0.015		
Acute myocardial infarction																
Diabetes		ns				ns				ns				ns		
Other heart disease		ns				ns				ns			0.12	0.040		
Age		ns		ns		ns		ns	0.22	0.000		ns	0.33	0.000	0.25	0.000
Sex	−0.23	0.001	−0.24	0.001		ns		ns		ns	−0.10	0.039		ns	0.10	0.047
			F = 6.1, 6.4 ^$^, 7.5 ^#^; *p* = 0.000, Adjust R^2^ = 0.21, 0.18 ^$^, 0.18 ^#^				F = 7.7, 4.5 ^$^, 4.8 ^#^; *p* = 0.000, Adjust R^2^ = 0.27, 0.09 ^$^, 0.08 ^#^				F = 23.9, 42.9 ^$^; *p* = 0.000; Adjust R^2^ = 0.32, 0.30 ^$^				F = 36.5, 42.2 **^$^**; *p* = 0.000; Adjust R^2^ = 0.38, 0.36 ^$^	

* Variables included in analyses are shown by numerical or textual entries. Empty fields show nonsignificant variables not included in a model. **^$^** Model w/o metals. ^#^ Model w/o metals and Si. CLT, clot lysis time; Abs_max_, maximum absorbance at 335 nm; LDL-C, low-density lipoprotein cholesterol; HDL-C, high-density lipoprotein cholesterol; GFR, glomerular filtration rate; BMI, body mass index; CVD, cardiovascular disease; AMI, acute myocardial infarction. NA, not available, ns, nonsignificant. Gray font indicates nonsignificant variables.

**Table 4:** Metallic and nonmetallic determinants of ischemic stroke.

Variable	Bivariate Correlations		Logistic Regression *							
			Model 1		Model 2		Model 3		Model 4	
	β	*P*	B	*P*	B	*P*	B	*P*	B	*P*
**Li**	−0.09	**0.032**	0.09	**0.015; 0.017** ^$^	0.12	0.068; **0.018** ^#^				
**Na**	−0.69	**0.000**	−0.06	**0.000**	−0.09	**0.000**				
**K**	−0.15	**0.000**		ns	−0.03	**0.007;** 0.151 ^#^				
**Ca**	−0.50	**0.000**	−0.09; −0.12 ^$^	0.059; **0.010** ^$^	−0.17	**0.027**				
**Sr**	0.19	**0.000**	0.03	**0.027; 0.021** ^$^	0.05	**0.033**				
**Ni**	−0.31	**0.000**	−0.01	**0.042; 0.022** ^$^	−0.02	**0.001**				
**Be**	0.15	**0.000**		ns	0.32	**0.035**				
**Al**	−0.11	**0.008**		ns	−0.01	**0.009;** 0.091 ^#^				
Mg	−0.15	**0.000**		ns		ns				
Fe	−0.25	**0.000**		ns		ns				
Cu	−0.17	**0.000**		ns		ns				
Zn	−0.16	**0.000**		ns		ns				
**Si**	−0.47	**0.000**	0.00	**0.006; 0.004** ^$^	0.00	**0.022**			−0.01	**0.000**
**B**	0.06	0.187	0.00	**0.048**; 0.098 ^$^	0.00	**0.023**				ns
P	−0.12	**0.004**		ns		ns				ns
S	0.03	0.529		ns		ns				ns
Fibrin CLT	0.15	**0.001**		ns		ns		ns		ns
Fibrin Abs_max_	0.21	**0.000**		ns		ns	7.06	**0.033**	9.61	**0.026**
Glucose	0.24	**0.000**				ns	0.14	0.130		ns
**LDL−C**	−0.19	**0.000**			−0.02	**0.048**	−0.01	**0.008**	−0.01	**0.015**
**HDL−C**	−0.28	**0.000**			−0.03	**0.001**	−0.01	**0.123**	−0.01	**0.030**
TG	0.12	**0.006**				ns		ns	0.01	0.079
***APOE112***	−0.00	0.456			1.94	**0.005**		ns		ns
GFR	−0.44	**0.000**			−0.11	**0.006**	−0.03	**0.006**	−0.06	**0.000**
Early CAD	0.47	**0.000**			3.49	**0.008**	1.30	**0.029**	0.94	**0.176**
Early MI	0.19	**0.000**			2.51	0.075		ns		ns
Hypertension	0.51	**0.000**			1.54	**0.034**	1.05	**0.001**	1.17	**0.002**
Diabetes	0.31	**0.000**				ns		ns		ns
Other heart disease	0.28	**0.000**			4.07	**0.000**	0.68	0.137	1.05	0.052
**Age**	0.52	**0.000**	0.11	**0.000**	0.08	**0.012**	**0.06**	**0.000**	0.07	**0.000**
Sex	0.16	**0.000**	0.97	**0.036;** 0.051 ^$^		ns	0.82	**0.010**	0.61	0.111
			−2 log likelihood = 194.9; Cox and Snell R^2^ = 0.61; Nagelkerke R^2^ = 0.82; % Correct 91.0		−2 log likelihood = 97.6; Cox and Snell R^2^ = 0.67; Nagelkerke R^2^ = 0.91; % Correct 96.0		−2 log likelihood = 339.1; Cox and Snell R^2^ = 0.44; Nagelkerke R^2^ = 0.60; % Correct 84.3		−2 log likelihood = 241.0; Cox and Snell R^2^ = 0.55; Nagelkerke R^2^ = 0.75; % Correct 88.3	

* Variables included in each model are shown by numerical or textual entries. Ischemic stroke was coded 1, no stroke 0. **^$^** Model w/o Abs_max_ and CLT. ^#^ Model w/o *APOE112*. Abs_max_, clot maximal absorbance at 340 nm; CLT, clot lysis time. LDL, low-density lipoprotein; HDL, high-density lipoprotein; GFR, glomerular filtration rate; CAD coronary artery disease; AMI, acute myocardial infarction. **Bold** font highlights significant variables.

**Table 5:** Lithium, sodium, calcium, and aluminum abrogate associations of fibrin Abs_max_ with ischemic stroke.

Variable	Logistic Regression *											
	Model 5		Model 6, -Li, -Na, -Ca, -Al		Model 7, +Li		Model 8, +Na		Model 9, +Ca		Model 10, +Al	
	β	*P*	B	*P*	B	*P*	B	*P*	B	*P*	B	*P*
Li	0.12	**0.068**			−0.05	0.141						
Na	−0.09	**0.000**					−0.08	**0.000**				
K	−0.03	**0.007**	−0.02	**0.002**	−0.02	**0.003**		ns		ns	−0.02	**0.016**
Ca	−0.17	**0.027**							−0.34	**0.000**		
Sr	0.05	**0.033**	0.04	**0.019**	0.04	**0.013**	0.04	**0.027**	0.05	**0.006**	0.03	**0.031**
Ni	−0.02	**0.001**	−0.02	**0.001**	−0.02	**0.001**	−0.02	**0.001**	−0.02	**0.001**	−0.02	**0.001**
Be	0.32	**0.035**	0.25	**0.007**	0.25	**0.007**			0.40	**0.000**	0.34	**0.001**
Al	−0.01	**0.009**									−0.01	**0.008**
Mg		ns		ns		ns		ns		ns		ns
Fe		ns	0.00	**0.033**	0.00	**0.034**	0.00	**0.046**		ns		ns
Cu		ns		ns		ns		ns		ns		ns
Zn		ns		ns		ns		ns		ns		ns
Si	0.00	**0.022**	−0.01	**0.000**	−0.01	**0.000**	0.00	**0.001**	0.00	**0.000**	−0.01	**0.000**
B	0.00	**0.023**		ns		ns	0.00	**0.026**		ns		ns
P		ns		ns		ns		ns		ns		ns
S		ns		ns		ns		ns	0.02	0.000		ns
Fibrin CLT		ns		ns		ns		ns		ns		ns
Fibrin Abs_max_		ns	10.72	**0.037**	8.89	0.094	−1.85	0.796	4.14	0.489	8.30	0.110
	−2 log likelihood = 97.8; Cox and Snell R^2^ = 0.67; Nagelkerke R^2^ = 0.91; % Correct 96.0		−2 log likelihood = 199.2; Cox and Snell R^2^ = 0.59; Nagelkerke R^2^ = 0.80; % Correct 90.8		−2 log likelihood = 196.8; Cox and Snell R^2^ = 0.59; Nagelkerke R^2^ = 0.81; % Correct 90.8		−2 log likelihood = 130.3; Cox and Snell R^2^ = 0.65; Nagelkerke R^2^ = 0.88; % Correct 95.2		−2 log likelihood = 154.6; Cox and Snell R^2^ = 0.63; Nagelkerke R^2^ = 0.86; % Correct 93.9		−2 log likelihood = 197.5; Cox and Snell R^2^ = 0.59; Nagelkerke R^2^ = 0.81; % Correct 91.	

* Variables included in each model are shown by numerical entries. Ischemic stroke was coded as 1, no stroke as 0. Adjusted for age, sex, triglycerides, LDL, HDL, glucose, GFR, earlier CAD, MI, hypertension, diabetes, other cardiovascular diseases, and medication use. CLT, clot lysis time. Abs_max_, clot maximal absorbance at 340 nm. **Bold** font highlights significant variables.

**Table 6:** R^2^ values and the risk of stroke associated with individual elements.

	Cox and Snell		Nagelkerke		Avg Risk, %
	R^2^	Stroke Risk *, %	R^2^	Stroke Risk *, %	
Model 1	63		84		
-Ni	63	<1	83	1	<1
-Ca	63	<1	84	<1	<1
-Li	63	<1	84	<1	<1
-Na	57	6	76	8	7
-Sr	62	1	83	1	1
-metals	49	14	66	18	16
-B	63	<1	84	<1	<1
-Si	62	1	82	2	1.5
-nonmetals	61	2	82	2	2
Model 2	67		90		
-Ni	66	1	90	<1	<1
-Ca	66	1	90	<1	<1
-Li	66	1	90	<1	<1
-Na	62	3	85	5	4
-K	66	1	90	<1	<1
-Be	66	1	90	<1	<1
-Al	66	1	89	1	1
-Sr	66	1	89	1	1
-metals	55	12	75	15	13.5
-B	66	1	90	<1	<1
-Si	65	2	89	1	1.5
-nonmetals	65	2	89	1	1.5

* Calculated as a difference between R^2^ values for the complete model and a model without an element.

## Data Availability

All data generated or analyzed during this study are included in this published article and Supplementary Materials.

## Supplementary Materials

The following supporting information can be downloaded at: https://www.mdpi.com/article/10.3390/life14050634/s1, Figure S1: Illustration of clotting and lysis variables; Figure S2: Pearsons’s correlations between plasma metallic/nonmetallic elements and fibrin clot properties in healthy controls and stroke patients. Figure S3. Relationships between serum elements and age in healthy controls and stroke patients. Figure S4. Relationships between serum elements and glomerular filtration rate (GFR) in healthy controls and stroke patients. Table S1: Settings of ICP instruments used for the quantification of metallic/non-metallic elements in serum; Table S2: ICP-MS isotopes and ICP-OES emission lines, LOD, and LOQ values for quantification of metallic and non-metallic elements; Table S3: Correlations between turbidimetric clotting and lysis variables in stroke patients and healthy individuals; Table S4: Descriptive statistics of the variables analyzed in the present study. Table S5: Correlations between metallic/nonmetallic elements in healthy controls and ischemic stroke patients: coefficients and P values.
