# Supplementary material for: Association of Metallic and Nonmetallic Elements with Fibrin Clot Properties and Ischemic Stroke
Source: Life (Basel). 2024 May 16;14(5):634. doi: 10.3390/life14050634 (PMC11122299; doi:10.3390/life14050634)
Supplement: Supplementary file 1 [file life-14-00634-s001.zip › life-2984923-supplementary.pdf]

Association of Metallic and Nonmetallic Elements with Fibrin Clot Properties and Ischemic Stroke

Hieronim Jakubowski [1,2,*], Marta Sikora [3], Ewa Bretes [2], Joanna Perła-Kaján [2], Olga Utyro [2], Izabela Wojtasz [4], Radosław Kaźmierski [5,6], Marcin Frankowski [7] and Anetta Zioła-Frankowska [7]

Supporting Information

Figure S1. Illustration of clotting and lysis variables. Variables examined in the present study, fibrin CLT and fibrin Abs_{max}, are highlighted in bold. Reproduced with permission from Sikora et al., PlosOne 2022.

Figure S2. Pearsons's correlations between plasma metallic/nonmetallic elements and fibrin clot properties in healthy controls and stroke patients. Zinc (A-D), calcium (E-H), beryllium (I, J), and sulfur (K-N). CLT (A, C, E, G, I, J, K, M) and Abs_{max} (B, D, F, H, L, N). Healthy controls (A, B, E, F, I, K, L) and stroke patients (C, D, G, H, J, M, N). Higher values of CLT and Abs_{max} usually indicate lower susceptibility to lysis and worse clot structure, respectively.

Figure S3. Relationships between serum elements and age in healthy controls and stroke patients. Levels of sodium (A, B), lithium (C, D), copper (E, F), iron (G, H), beryllium (I, J), aluminum (K, L), silicon (M, N) and strontium (O, P) are plotted vs. age in healthy individuals (A, C, E, G, I, K, M, O) and stroke patients (B, D, F, H, J, L, N, P).

Figure S4. Relationships between serum elements and glomerular filtration rate (GFR) in healthy controls and stroke patients. Levels of sodium (A, B), potassium (C, D), lithium (E, F), strontium (G, H), copper (I, J), aluminum (K, L),and silicon (M, N) are plotted vs. GFR in healthy individuals (A, C, E, G, I, K, M) and stroke patients (B, D, F, H, J, L, N).

Table S1.

Table S3. Correlations between turbidimetric clotting and lysis variables in stroke patients and healthy individuals.

Table S4. Descriptive statistics of the variables analyzed in the present study.

Table S5. Correlations between fibrin clot properties vs. metals in stroke patients and healthy individuals.

Table S5. R^2 values and the risk of ischemic stroke associated with individual elements.

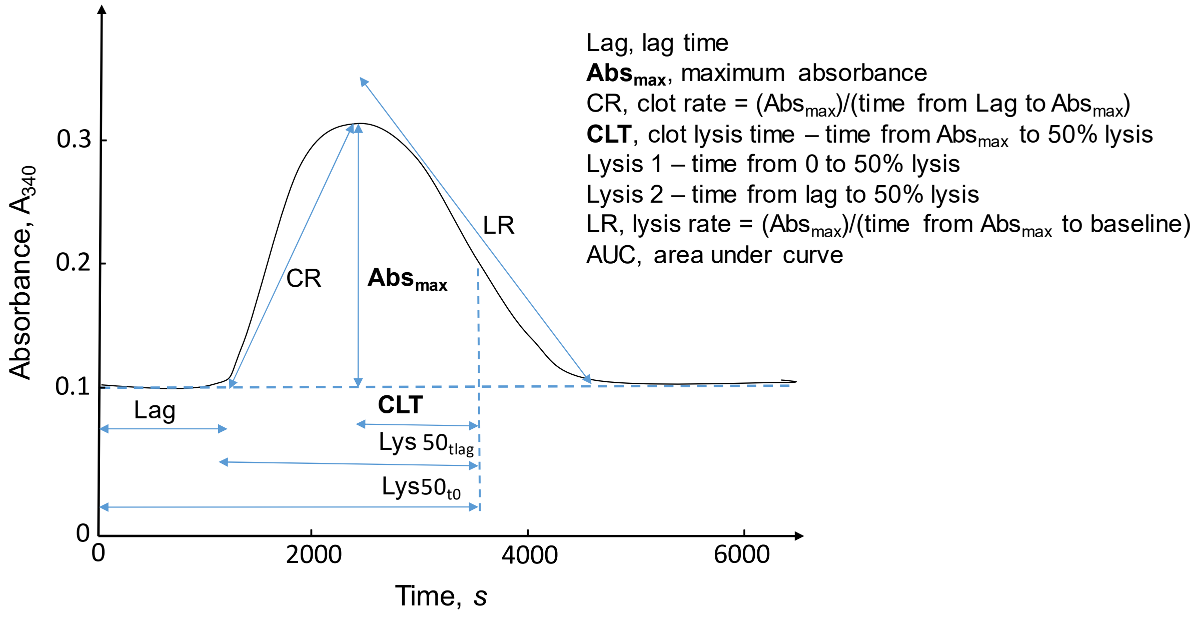

Lag, lag time
Absmax, maximum absorbance
CR, clot rate = (Absmax)/(time from Lag to Absmax)
CLT, clot lysis time – time from Absmax to 50% lysis
Lysis 1 – time from 0 to 50% lysis
Lysis 2 – time from lag to 50% lysis
LR, lysis rate = (Absmax)/(time from Absmax to baseline)
AUC, area under curve
Absorbance, A340
Time, s
0.3
0.2
0.1
0
0
2000
4000
6000
CR
Absmax
LR
Lag
CLT
Lys 50tlag
Lys50t0

Figure S1.

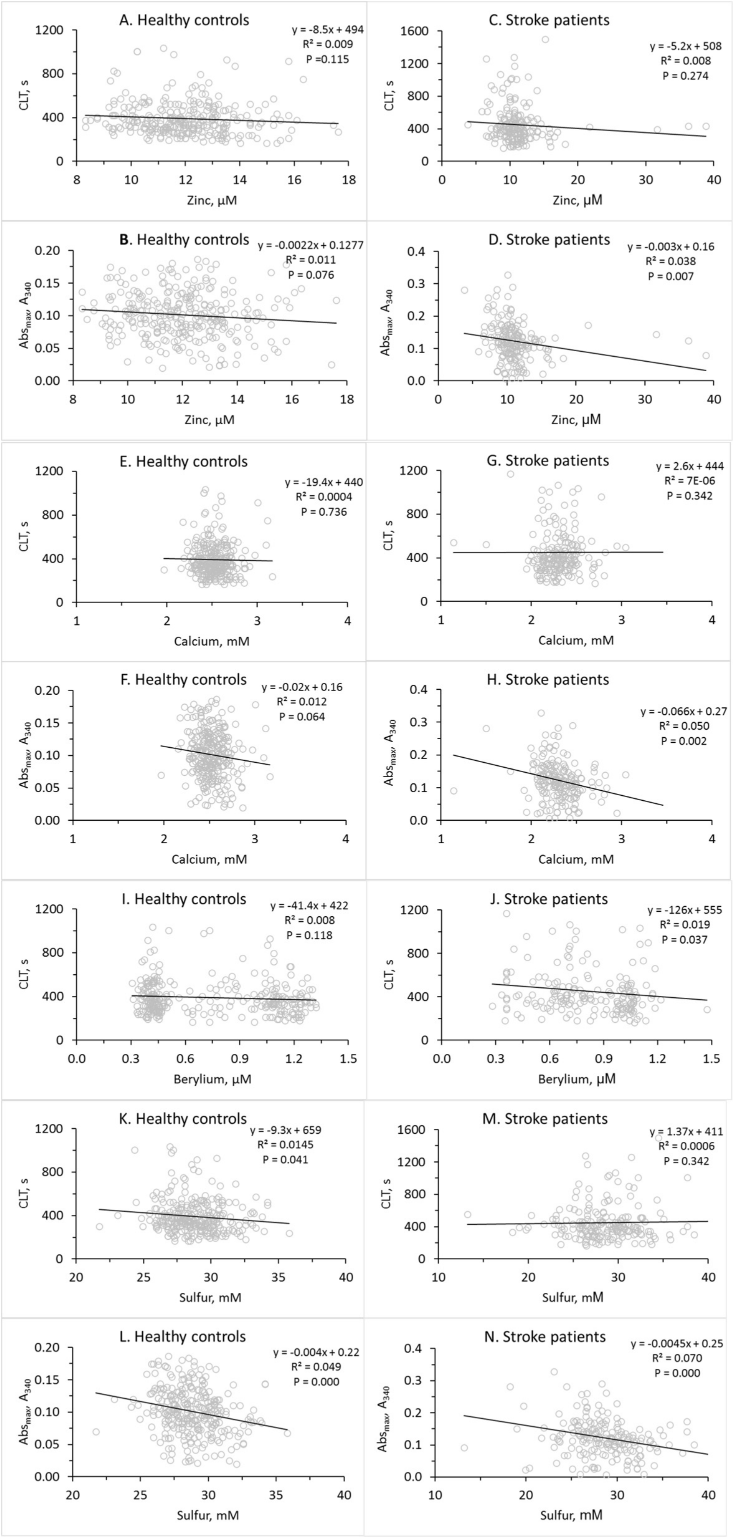
A. Healthy controls
y = -8.5x + 494
R² = 0.009
P =0.115
CLT, s
Zinc, µM
C. Stroke patients
y = -5.2x + 508
R² = 0.008
P = 0.274
CLT, s
Zinc, µM
B. Healthy controls
y = -0.0022x + 0.1277
R² = 0.011
P = 0.076
Absmax, A340
Zinc, µM
D. Stroke patients
y = -0.003x + 0.16
R² = 0.038
P = 0.007
Absmax, A340
Zinc, µM
E. Healthy controls
y = -19.4x + 440
R² = 0.0004
P = 0.736
CLT, s
Calcium, mM
G. Stroke patients
y = 2.6x + 444
R² = 7E-06
P = 0.342
CLT, s
Calcium, mM
F. Healthy controls
y = -0.02x + 0.16
R² = 0.012
P = 0.064
Absmax, A340
Calcium, mM
H. Stroke patients
y = -0.066x + 0.27
R² = 0.050
P = 0.002
Absmax, A340
Calcium, mM
I. Healthy controls
y = -41.4x + 422
R² = 0.008
P = 0.118
CLT, s
Berylium, µM
J. Stroke patients
y = -126x + 555
R² = 0.019
P = 0.037
CLT, s
Berylium, µM
K. Healthy controls
y = -9.3x + 659
R² = 0.0145
P = 0.041
CLT, s
Sulfur, mM
M. Stroke patients
y = 1.37x + 411
R² = 0.0006
P = 0.342
CLT, s
Sulfur, mM
L. Healthy controls
y = -0.004x + 0.22
R² = 0.049
P = 0.000
Absmax, A340
Sulfur, mM
N. Stroke patients
y = -0.0045x + 0.25
R² = 0.070
P = 0.000
Absmax, A340
Sulfur, mM

Figure S2A-M

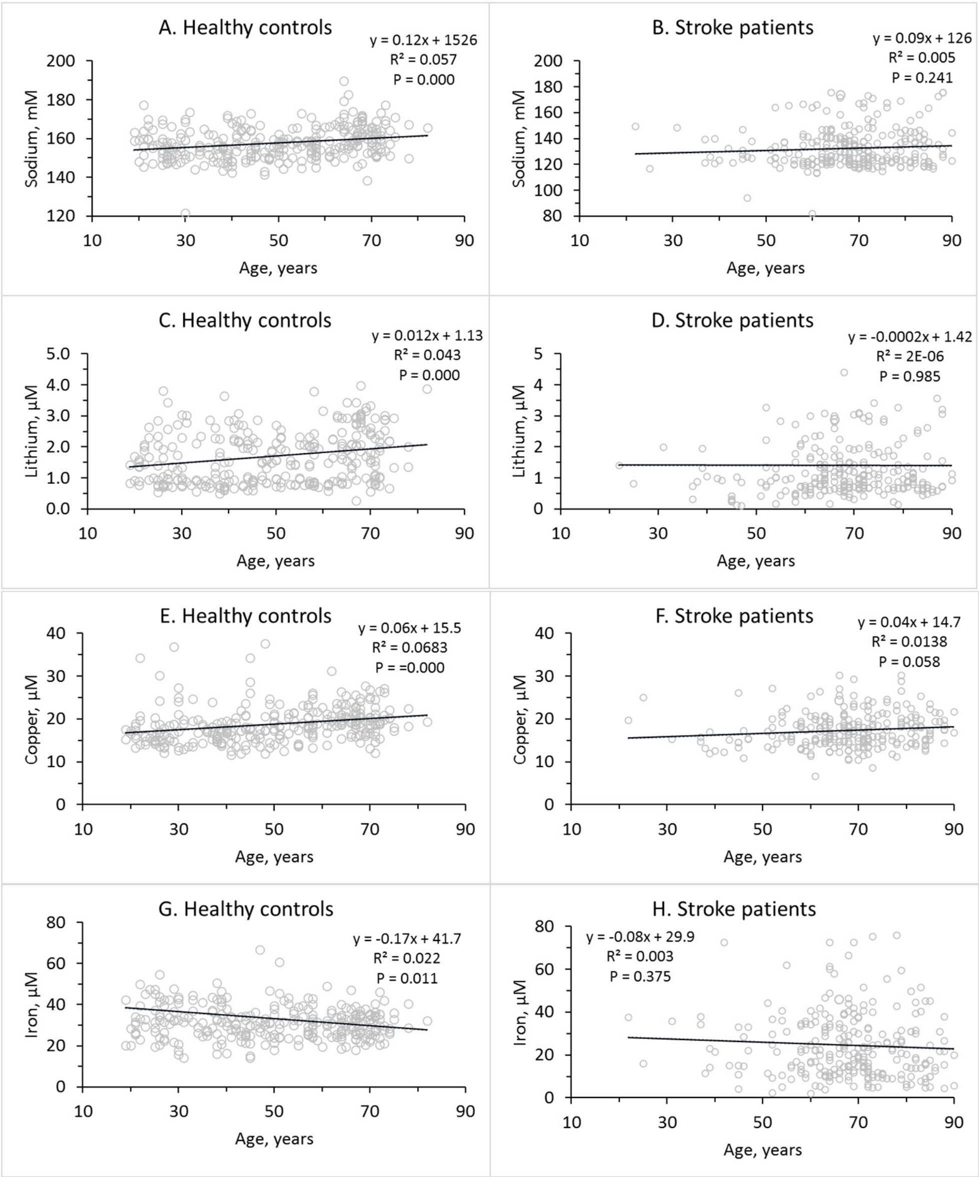
A. Healthy controls
y = 0.12x + 1526
R² = 0.057
P = 0.000
Sodium, mM
Age, years
B. Stroke patients
y = 0.09x + 126
R² = 0.005
P = 0.241
Sodium, mM
Age, years
C. Healthy controls
y = 0.012x + 1.13
R² = 0.043
P = 0.000
Lithium, µM
Age, years
D. Stroke patients
y = -0.0002x + 1.42
R² = 2E-06
P = 0.985
Lithium, µM
Age, years
E. Healthy controls
y = 0.06x + 15.5
R² = 0.0683
P = =0.000
Copper, µM
Age, years
F. Stroke patients
y = 0.04x + 14.7
R² = 0.0138
P = 0.058
Copper, µM
Age, years
G. Healthy controls
y = -0.17x + 41.7
R² = 0.022
P = 0.011
Iron, µM
Age, years
H. Stroke patients
y = -0.08x + 29.9
R² = 0.003
P = 0.375
Iron, µM
Age, years

Figure S3A-H

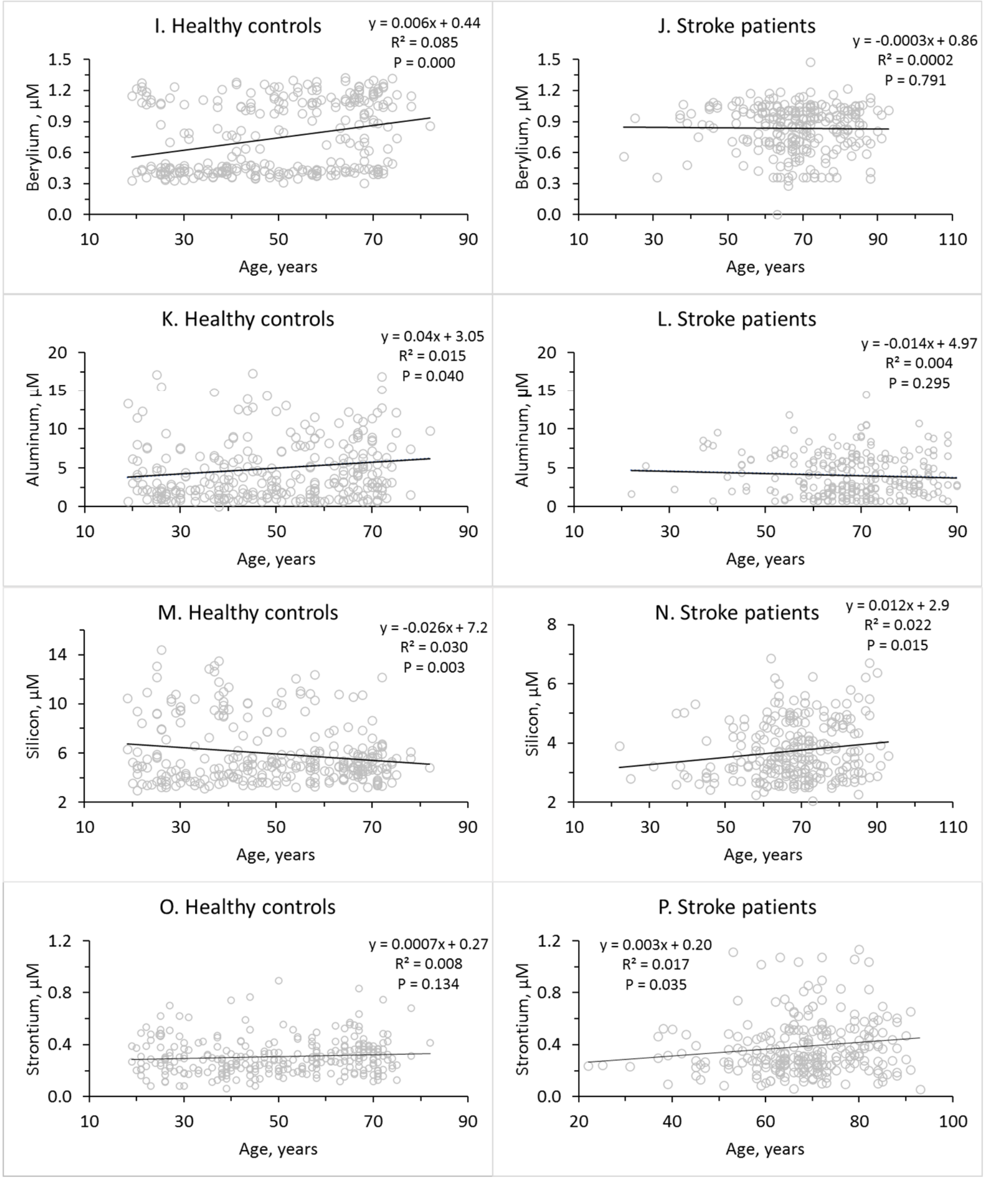

I. Healthy controls
y = 0.006x + 0.44
R² = 0.085
P = 0.000
Berylium, µM
Age, years
J. Stroke patients
y = -0.0003x + 0.86
R² = 0.0002
P = 0.791
Berylium, µM
Age, years
K. Healthy controls
y = 0.04x + 3.05
R² = 0.015
P = 0.040
Aluminum, µM
Age, years
L. Stroke patients
y = -0.014x + 4.97
R² = 0.004
P = 0.295
Aluminum, µM
Age, years
M. Healthy controls
y = -0.026x + 7.2
R² = 0.030
P = 0.003
Silicon, µM
Age, years
N. Stroke patients
y = 0.012x + 2.9
R² = 0.022
P = 0.015
Silicon, µM
Age, years
O. Healthy controls
y = 0.0007x + 0.27
R² = 0.008
P = 0.134
Strontium, µM
Age, years
P. Stroke patients
y = 0.003x + 0.20
R² = 0.017
P = 0.035
Strontium, µM
Age, years

Figure S3I-P

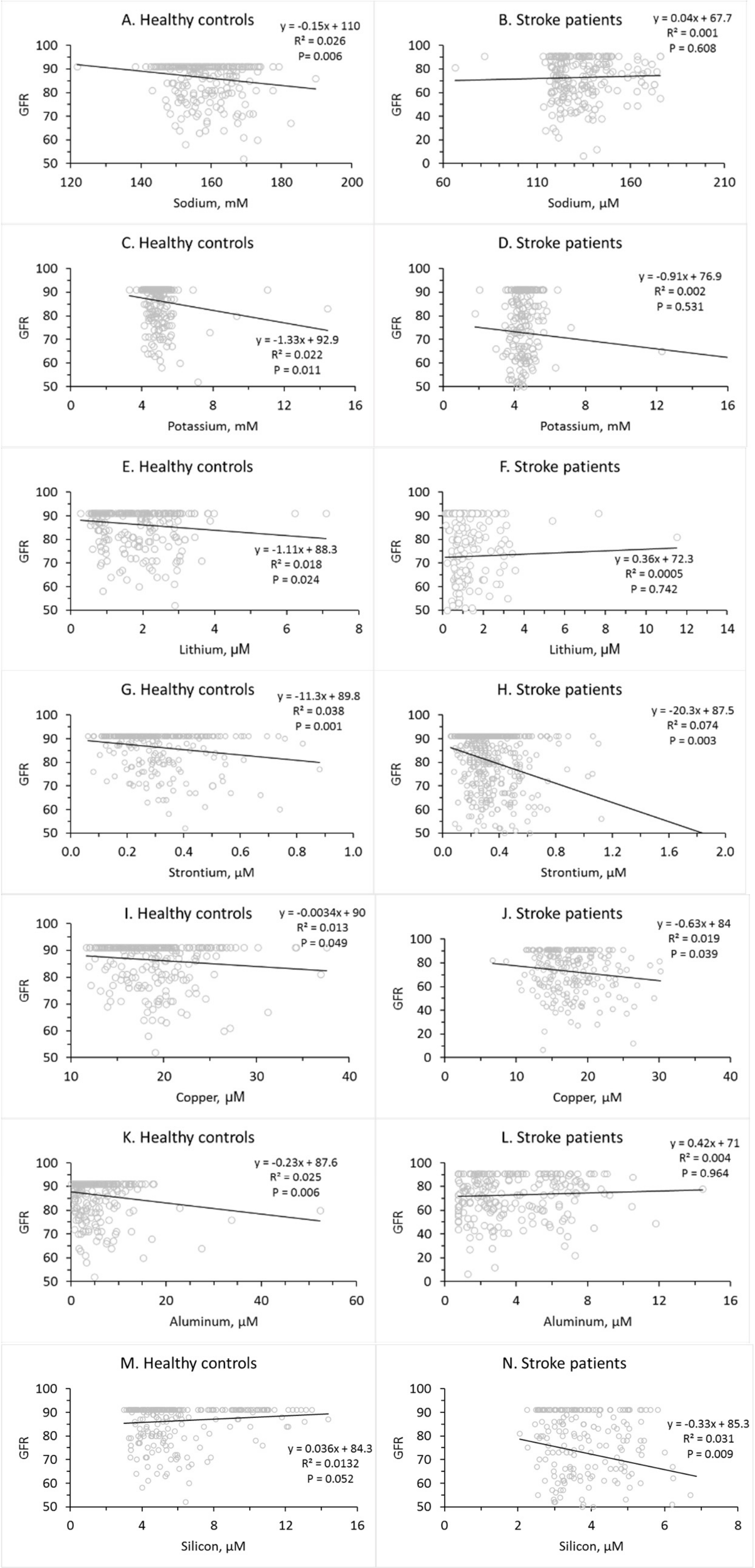
A. Healthy controls
y = -0.15x + 110
R² = 0.026
P= 0.006
GFR
Sodium, mM
B. Stroke patients
y = 0.04x + 67.7
R² = 0.001
P = 0.608
GFR
Sodium, µM
C. Healthy controls
y = -1.33x + 92.9
R² = 0.022
P = 0.011
GFR
Potassium, mM
D. Stroke patients
y = -0.91x + 76.9
R² = 0.002
P = 0.531
GFR
Potassium, mM
E. Healthy controls
y = -1.11x + 88.3
R² = 0.018
P = 0.024
GFR
Lithium, µM
F. Stroke patients
y = 0.36x + 72.3
R² = 0.0005
P = 0.742
GFR
Lithium, µM
G. Healthy controls
y = -11.3x + 89.8
R² = 0.038
P = 0.001
GFR
Strontium, µM
H. Stroke patients
y = -20.3x + 87.5
R² = 0.074
P = 0.003
GFR
Strontium, µM
I. Healthy controls
y = -0.0034x + 90
R² = 0.013
P = 0.049
GFR
Copper, µM
J. Stroke patients
y = -0.63x + 84
R² = 0.019
P = 0.039
GFR
Copper, µM
K. Healthy controls
y = -0.23x + 87.6
R² = 0.025
P = 0.006
GFR
Aluminum, µM
L. Stroke patients
y = 0.42x + 71
R² = 0.004
P = 0.964
GFR
Aluminum, µM
M. Healthy controls
y = 0.036x + 84.3
R² = 0.0132
P = 0.052
GFR
Silicon, µM
N. Stroke patients
y = -0.33x + 85.3
R² = 0.031
P = 0.009
GFR
Silicon, µM

Figure S4A-N

**Table S1.** Settings of ICP instruments used for the quantification of metallic/non-metallic elements in serum.			
**Elements**	**Analytical techniques**		**ICP settings**
Ca, Mg, Na, K, P, S	Inductively coupled plasma optical emission spectrometry (ICP-OES)	Inductively coupled plasma optical emission spectrometer (ICP-OE 9820, Shimadzu, Japan)	Radio frequency power generator: 1.2 kW; gas: argon; plasma gas flow rate: 9.00 L min^{-1}; auxiliary gas flow rate: 0.6 L min^{-1}; nebulization gas flow rate: 0.7 L min^{-1}, plasma view: vertical torch, axial view; torch: mini-torch (quartz); nebulizer: coaxial; chamber: cyclone (glass); spray chamber temperature: room temperature; drain: gravity fed; injector tube: quartz (1.2 mm i.d.); background correction: 2-points; exposure time: 30 s
Al, Be, Cu, Fe, Ni, Li, Sr, Zn, B, Si	Inductively coupled plasma mass spectrometry (ICP-MS)	Inductively coupled plasma mass spectrometer (ICP-MS 2030, Shimadzu, Japan)	Radio frequency power generator: 1.2 kW; gas: argon; plasma gas flow rate: 8.0 L min^{-1}; auxiliary gas flow rate: 1.1 L min^{-1}; nebulization gas flow rate: 0.7 L min^{-1}; torch: mini-torch (quartz); nebulizer: coaxial; chamber: cyclone (glass); spray chamber temperature: 5°C; drain: gravity fed; internal standard: automatic addition; sampling depth: 5 mm; collision cell gas flow: 6.5 mL min^{-1} (He); cell voltage: −21 V, energy filter: 7.0 V.

**Table S2.** ICP-MS isotopes and ICP-OES emission lines, LOD, and LOQ values for quantification of metallic and non-metallic elements			
**Element**	**Isotope or wavelength** (nm)*	**LOD** (µg/L)	**LOQ** (µg/L)
Ca	396.8*	0.572	1.907
Mg	279.5*	0.035	0.117
Na	589.6*	0.044	0.146
K	766.5*	1.662	5.541
P	178.3*	31.13	103.7
S	182.6*	3.164	10.54
Al	27	0.541	1.803
B	11	1.309	4.365
Ba	138	0.104	0.347
Be	9	0.008	0.028
Cu	63	0.051	0.170
Fe	56	0.041	0.137
Ni	58	0.022	0.073
Li	7	0.068	0.265
Si	28	1.126	4.321
Sr	88	0.047	0.157
Zn	66	0.036	0.120

Table S3. Correlation coefficients for relationships between turbidimetric clotting and lysis variables in stroke patients and healthy individuals*.

	Clotting/lysis correlation coefficients						
Variable	MaxAbs, Abs_{max}*	Clot Rate	$Lys50_{t0}$	$Lys50_{tlag}$	$Lysis50_{MA}$, CLT*	Lysis Rate	Lysis Area AUC
	Stroke patients (n = 191)						
Lag	-0.61	-0.65	0.45	-0.02 (*P*=0.801)	-0.01 (*P*=0.929)	0.44	0.41
AbsMax, Abs_{max}*		0.89	-0.10 (*P*=0.183)	0.21 (*P*=0.003)	0.29	-0.77	0.88
Clot Rate			-0.45	-0.16 (*P*=0.026)	0.01 (*P*=0.837)	-0.72	0.61
$Lys50_{t0}$				0.89	0.86	-0.07 (*P*=0.204)	0.65
$Lys50_{tlag}$					0.75	-0.05 (*P*=0.553)	0.51
$Lysis50_{MA}$, CLT*						-0.05 (*P*=0.527)	0.58
Lysis Rate							-0.54
	Healthy controls (n = 291)						
Lag	-0.60	-0.51	0.17 (*P*=0.003)	-0.29	-0.14 (*P*=0.013)	0.44	0.42
AbsMax, Abs_{max}*		0.88	-0.32	0.60	0.55	-0.70	0.88
Clot Rate			0.00 (*P*=0.982)	0.25	0.35	-0.70	0.64
$Lys50_{t0}$				0.89	0.86	-0.07 (*P*=0.204)	0.65
$Lys50_{tlag}$					0.91	-0.27	0.82
$Lysis50_{MA}$, CLT*						-0.28	0.79
Lysis Rate							-0.48

*Nomenclature is after Carter *et al.*, *Arterioscler Thromb Vasc Biol* 2007; 27:2783-2789. The clotting and lysis variables are illustrated in **Figure S1**. Terms Abs_{max} and CLT are used in the present study and refer to terms MaxAbs and $Lysis50_{MA}$, respectively, of Carter *el al.* ATVB 2007. *P* values for these correlations were <0.000 except where written otherwise.

**Table S4.** Descriptive statistics of the variables analyzed in the present study.			
**Variable**	**Stroke patients**	**Healthy controls**	***P*-value**
Fibrin clot properties			
	(n = 191)	(n = 291)	
Fibrin Abs_{max}, A_{340}	0.123±0.065	0.101±0.036	0.000
Fibrin CLT, s	450±225	391±154	0.001
Metals			
	(n = 260)	(n = 291)	
Fe, µM	24.3±17.3	33.3±19.0	0.000
Cu, µM	17.3±4.0	18.7±4.1	0.000
Zn, µM	11.1±3.6	12.0±1.7	0.000
Ni, µM	0.31±0.55	0.62±0.37	0.000
Ca, mM	2.29±0.21	2.52±0.16	0.000
Sr, µM	↑0.39±0.25	0.31±0.14	0.000
Mg, mM	0.83±0.10	0.86±0.06	0.000
Li, µM	1.40±1.92	1.70±0.93	0.018
Na, mM	131±17	158±8	0.000
K, mM	4.66±1.50	4.83±0.72	0.000
Be, µM	↑0.84±0.22	0.74±0.34	0.000
Al, µM	4.02±2.65	4.96±5.22	0.007
B, µM	37.7±117	26.7±72.5	0.247
P, µM	3.69±0.76	3.85±0.49	0.004
S, mM	28.9±3.6	28.8±2.0	0.520
Si, µM	3.73±097	5.93±2.47	0.000
Nonmetals			
Plasma creatinine, µM	↑87.7±33.6	70.5±13.2	0.000
Plasma glucose, mM	↑6.6±2.7	5.6±0.7	0.000
Total cholesterol, mM	179±49	208±40	0.000
LDL cholesterol, mM	104±42	119±37	0.000
HDL cholesterol, mM	52±28	66±18	0.000
Triglycerides, mM	↑131±68	114±79	0.007
Established risk factors			
BMI, kg/m^2	NA	26.3±4.7	
GFR, mL/min/1.73 m^2	72.8±18.0	86.4±7.8	0.000
Earlier CVD, %	23.3	2.3	0.000
Earlier MI #6, %	8.7	0.7	0.000
Other heart disease, %	22.5	4.3	0.000
Hypertension, %	77.5	21.7	0.000
Diabetes, %	24.0	3.7	0.000
Smoking, %	47.5	NA	
Medications, %	78.3	7.7%	0.000
Age, years	68±12	50±17	0.000
Female sex, %	45.0	59.7	0.000

GFR, glomerular filtration rate; BMI, body mass index; CVD, cardiovascular disease; LDL, low-density lipoprotein; HDL. High-density lipoprotein; CLT, clot lysis time; Abs_{max}, maximum absorbance at 335 nm; NA, not available. Up arrows ↑ indicate variables that were increased in ischemic stroke patients.

Table S5. Correlations between elements in healthy controls and ischemic stroke patients: coefficients and *P*-values.

Element	Ni	Cu	Zn	Ca	Sr	Mg	Li	Na	K	Be	Al	B	P	S	Si
	**Healthy controls** (n = 291)														
Fe #45	ns	-.14 P=.016	ns	ns	ns	ns	ns	ns	ns	ns	ns	ns	ns	.21 P=.000	.12 P=.037
Ni #48		.17 P=.003	ns	.19 P=.001	ns	ns	ns	ns	.12 P=.038	ns	**.29 P=.000**	ns	ns	ns	ns
Cu #46			.14 P=.014	**.28 P=.000**	.13 P=.029	**ns**	**.25 P=.000**	**.31 P=.000**	ns	**.31 P=.000**	**.21 P=.000**	ns	.37 P=.000	-.14 P=.020	ns
Zn #47				**.51 P=.000**	.19 P=.001	**.36 P=.000**	**.26 P=.000**	**.32 P=.000**	ns	**.52 P=.000**	**.22 P=.000**	.14 P=.019	ns	.18 P=.002	ns
Ca #49					**.23 P=.000**	**.44 P=.000**	**.17 P=.002**	**.60 P=.000**	.12 P=.032	**.36 P=.000**	.15 0.012	ns	.29 P=.000	.51 P=.000	ns
Sr #69						**.13 P=.020**	**.35 P=.000**	ns	ns	**.27 P=.000**	.15 P=.011	ns	ns	ns	ns
Mg #50							ns	**.45 P=.000**	.15 P=.011	ns	ns	ns	**.24 P=.000**	**.37 P=.000**	ns
Li #51								**.32 P=.000**	ns	**.47 P=.000**	**.24 P=.000**	.14 P=.015	ns	**-.27 P=.000**	**-.23 P=.000**
Na #52									ns	**.33 P=.000**	.12 P=.044	ns	**.25 P=.000**	.19 P=.001	ns
K #53										ns	ns	ns	.13 P=.023	ns	ns
Be #55											**.30 P=.000**	.18 P=.001	ns	**-.28 P=.000**	**-.23 P=.000**
Al #58												ns	ns	-.18 P=.002	-.16 P=.007
B #54													ns	ns	ns
P #56														.15 P=.009	ns
S #57															**.26 P=.000**

* The strongest correlations are highlighted in **bold text**.

Table S3, continued.

Element	Ni	Cu	Zn	Ca	Sr	Mg	Li	Na	K	Be	Al	B	P	S	Si
	**Stroke patients*** (n = 262)														
Fe #45	**.21 P=.001**	ns	**.34 P=.000**	ns	ns	ns	ns	**.41 P=.000**	.21 P=.001	**-.45 P=.000**	**-.37 P=.000**	-.17 P=.006	.12 P=.046	-.17 P=.006	**.47 P=.000**
Ni #48		ns	ns	ns	ns	ns	ns	ns	ns	ns	ns	ns	ns	ns	ns
Cu #46			ns	**.25 P=.000**	.17 P=.007	ns	ns	**.32 P=.000**	ns	-.14 P=.020	ns	ns	ns	ns	**.27 P=.000**
Zn #47				**.24 P=.000**	ns	.18 P=.004	ns	.15 P=.017	ns	ns	ns	ns	.17 P=.007	**.25 P=.000**	ns
Ca #49					**.27 P=.000**	**.38 P=.000**	ns	**.43 P=.000**	**.26 P=.000**	.17 P=.007	ns	ns	**.42 P=.000**	**.65 P=.000**	.18 P=.003
Sr #69						ns	ns	.17 P=.005	ns	ns	ns	ns	ns	ns	**.30 P=.000**
Mg #50							ns	ns	ns	**.26 P=.000**	.18 P=.004	ns	**.28 P=.000**	**.48 P=.000**	ns
Li #51								**.24 P=.000**	ns	.15 P=.017	ns	ns	ns	ns	.14 P=.022
Na #52									.18 P=.003	**-.46 P=.000**	-.16 P=.011	ns	ns	-.14 P=.021	**.53 P=.000**
K #53										ns	ns	ns	ns	ns	.16 P=.009
Be #55											**.45 P=.000**	.17 P=.006	ns	**.46 P=.000**	**-.38 P=.000**
Al #58												.13 P=.041	ns	**.23 P=.000**	-.16 P=.007
B #54													ns	ns	ns
P #56														**.43 P=.000**	ns
S #57															**-.25 P=.000**

*Elements associated with stroke are highlighted in light green. Correlations changed in stroke are highlighted in yellow. The strongest correlations are highlighted in **bold text**.
